# Quantum-Size FeS_2_ with Delocalized Electronic Regions Enable High-Performance Sodium-Ion Batteries Across Wide Temperatures

**DOI:** 10.1007/s40820-025-01858-2

**Published:** 2025-07-29

**Authors:** Tianlin Li, Danyang Zhao, Meiyu Shi, Chao Tian, Jie Yi, Qing Yin, Yongzhi Li, Bin Xiao, Jiqiu Qi, Peng Cao, Yanwei Sui

**Affiliations:** 1 China University of Mining and Technology, Xuzhou, 221116 People’s Republic of China; 2 Jiangsu Province Engineering Laboratory of High-Efficient Energy Storage Technology and Equipment, School of Materials Science and Physics, China University of Mining and Technology, Xuzhou, 221116 People’s Republic of China; 3 Department of Chemical and Materials Engineering, University of Auckland, Auckland, 1142 New Zealand

**Keywords:** Quantum-size effect, Electron delocalization, Efficient short-range transfer kinetics, Wide-temperature, Sodium-ion batteries

## Abstract

Wide-temperature applications of sodium-ion batteries (SIBs) are severely limited by the sluggish ion insertion/diffusion kinetics of conversion-type anodes. Quantum-sized transition metal dichalcogenides possess unique advantages of charge delocalization and enrich uncoordinated electrons and short-range transfer kinetics, which are crucial to achieve rapid low-temperature charge transfer and high-temperature interface stability. Herein, a quantum-scale FeS_2_ loaded on three-dimensional Ti_3_C_2_ MXene skeletons (FeS_2_ QD/MXene) fabricated as SIBs anode, demonstrating impressive performance under wide-temperature conditions (− 35 to 65 °C). The theoretical calculations combined with experimental characterization interprets that the unsaturated coordination edges of FeS_2_ QD can induce delocalized electronic regions, which reduces electrostatic potential and significantly facilitates efficient Na^+^ diffusion across a broad temperature range. Moreover, the Ti_3_C_2_ skeleton reinforces structural integrity via Fe–O–Ti bonding, while enabling excellent dispersion of FeS_2_ QD. As expected, FeS_2_ QD/MXene anode harvests capacities of 255.2 and 424.9 mAh g^−1^ at 0.1 A g^−1^ under − 35 and 65 °C, and the energy density of FeS_2_ QD/MXene//NVP full cell can reach to 162.4 Wh kg^−1^ at − 35 °C, highlighting its practical potential for wide-temperatures conditions. This work extends the uncoordinated regions induced by quantum-size effects for exceptional Na^+^ ion storage and diffusion performance at wide-temperatures environment.

**Supplementary Information:**

The online version contains supplementary material available at 10.1007/s40820-025-01858-2.

## Highlights

- Quantum-scaled FeS_2_ induces delocalized electronic regions, effectively reducing electrostatic potential barriers and accelerating Na^+^ diffusion kinetics.
- The free charge accumulation regions were formed by edge mismatched atoms, activating numerous electrochemically sites to enable high-capacity Na^+^ storage and ultrafast-ion transport across wide temperature range (−35 to 65 °C).
- The FeS_2_ QD/MXene anode delivers superior wide-temperature capacity of 255.2 mAh g^−1^ (−35 °C) and 424.9 mAh g^−1^ (65 °C) at 0.1 A g^−1^. The FeS_2_ QD/MXene//NVP cell achieves a record energy density of 162.4 Wh kg⁻^1^ at − 35 °C.

**Supplementary Information:**

The online version contains supplementary material available at 10.1007/s40820-025-01858-2.

## Graphical abstract

**Figure:**
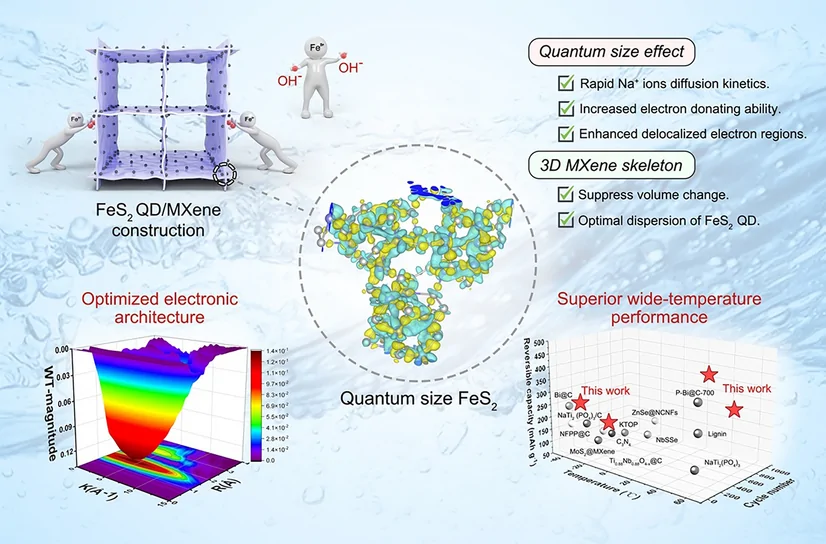

## Introduction

Sodium-ion batteries (SIBs) have emerged as a significant technological innovation, due to the abundance of sodium resources, superior safety features and lower cost, which can be promising alternative for lithium-ion batteries devices ascribing to their similar redox mechanisms [1, 2]. Although SIBs have exhibit dramatically improved electrochemical performance in recent years, the wide-temperature (− 35 to 65 °C) properties have also been the crucial bottleneck for expanding the application of SIBs, which are mainly hindered by poor ion conduction efficiency and reduced diffusion kinetics of electrodes [3–6]. Therefore, developing novel anodes with high capacity, long lifespan and fast-ion transport is essential to address bottlenecks in the development of wide-temperature SIBs [7].

Ferrous disulfide (FeS_2_), a prominent transition metal dichalcogenides (TMDs), demonstrates the sodium storage ability, coupling conversion and alloying mechanisms (FeS_2_ + 4Na^+^  + 4e^−^  ↔ Fe + 2Na_2_S) [8–10]. Furthermore, FeS_2_ is considered as a potential anode material due to abundant reserves, low cost and remarkable theoretical capacity (894 mAh g^−1^) [11, 12]. However, FeS_2_ electrodes typically exhibit sluggish Na^+^ diffusion barriers, poor reversibility and substantial volume changes during operation [13]. At low temperature (below − 25 °C), increased electrolyte viscosity impedes ion and charge transfer, resulting in slow reaction kinetics. In contrast, high-temperature (above 50 °C) condition enhances system energy, leading to more active interfacial side reactions and consequently resulting in severe capacity [14, 15].

Various strategies have been proposed to address these challenges including size regulation, defect fabrication and electronic optimization [16–20]. For instance, constructing the defective regions directionally (vacancies, doping, grain boundaries, etc.) can effectively regulate the electrochemical active sites at the atomic level, thereby enhancing Na^+^ storage capacity and conversion kinetics. Cao et al. demonstrated that confining SnS within N/S co-doped graphene could reduce the energy activation and enhance power density [16]. However, although N/S-doped carbon has shown improved electronic conductivity and increased active sites, undesirable defect concentrations and incomplete utilization of active materials still restrain the electrochemical performance to a high level. The direct introduction of defect states into active components is considered as a potential avenue to improve its defect concentration. Our group developed a porous conducting matrix confining defect-rich CoSe_0.5_S_1.5_ composite, the defect manufacturing strategy effectively optimizes charge distribution and electrochemical activity, the prepared CoSe_0.5_S_1.5_/GA anode could possess superior rate performance 288.2 mAh g^−1^ at 5 A g^−1^-based sodium-ion capacitor [17]. However, such defect engineering strategies primarily rely on chemical doping or vacancy creation, which inevitably compromise structural stability and limit accessible defect density. This intrinsic trade-off between defect concentration and stability poses a fundamental challenge for achieving wide-temperature adaptability. Therefore, wide-temperature (− 35 to 65 °C) electrode still remains a key issue.

Recent studies have established that edged uncoordinated atoms could effectively promote local electron delocalization, enhance interfacial charge interactions and improve the conversion reaction reversibility [18, 19]. Notably, quantum-size effects naturally maximize the proportion of edge uncoordinated atoms without relying on extrinsic defect engineering. Compared to conventional defect regulation, quantum confinement intrinsically amplifies charge delocalization through unsaturated coordination edges while maintaining structural robustness, a critical advantage for wide-temperature operation. In conclusion, we proposed an innovative strategy for constructing quantum-sized TMDs to maximize the edge atomic content. Nevertheless, TMDs with quantum sizes ranging from 5 to 10 nm tend to agglomerate during the ion insertion and diffusion process due to their high activity; hence, it is crucial to employ an appropriate carrier, which can achieve optimized dispersion and construct strong interfacial coupling of TMDs component. Carbonaceous materials such as activated carbon and graphene have captured extensive attention to serve as substrates to buffer volume change [20–22]. For instance, Zhou and colleagues developed a SIBs anode using SnS/SNC, and the SNC matrix not only achieves uniform dispersion of SnS but also effectively enhances the electrode stability during the electrochemical reaction process [22]. However, traditional carbon materials exhibit unsatisfactory capability in Na^+^ energy storage systems due to their inherent electrochemical reaction mechanisms.

Consequently, we have explored two-dimensional transition metal carbides and carbonitrides (MXene) as substrate, which can possess a graphene-like structure and deliver higher specific capacity. Ti_3_C_2_ MXene has garnered significant interest due to its excellent metal-level carrier transport efficiency and abundance of surface oxygen-containing functional groups, showing promising potential in the field of energy storage and catalysis applications [23, 24]. Furthermore, recent studies have demonstrated that Ti_3_C_2_ MXene can be gelatinization by divalent metal ions and form a three-dimensional porous skeleton, which enrich the active sites and restrict the self-stacking effect [25]. This robust 3D network is fabricated via the strong interaction between multivalent metal ions and MXene surface terminations and effectively alleviates the structure collapse, associated with TMD-based electrodes.

Herein, a hybrid of well-dispersed and uniform-loading FeS_2_ quantum dot embedded in three-dimensional Ti_3_C_2_ MXene skeleton (FeS_2_ QD/MXene) is fabricated. Compared with conventional well-crystallized TMDs materials, the existence of quantum-scale FeS_2_ could manipulate uncoordinated electronic states, supply ample reaction active sites and accelerate the charge transfer kinetic, which contributes to high-efficiency sodium storage under wide temperature. Density functional theory (DFT) calculations and X-ray absorption spectroscopy (XAS) analyses demonstrate that the FeS_2_ QD plays a crucial role in expanding charge delocalization regions, enhancing negative electrostatic potential and accelerating ions transport. Importantly, the robust interfacial coupling between FeS_2_ QD and MXene substrates could be beneficial to structural stability and electrolyte transportation process. Benefiting from these features, the prepared anode exhibited excellent temperature tolerance (255.2 and 424.9 mAh g^−1^ at 0.1 A g^−1^ under − 35 and 65 °C) and remarkable cycling stability (370.1 mAh g^−1^ at 1 A g^−1^ after 2500 cycles). In addition, the assembled FeS_2_ QD/MXene//NVP full cell presents a high energy density of 162.4 Wh kg^−1^ at 0.1C under − 35 °C. This quantum-scale-induced electronic property modulation strategy provides a new path for developing long-life and high-capacity SIBs anodes operated under wide-temperature conditions.

## Experimental Section

### Materials

Ferrous chloride (FeCl_2_), Ti_3_AlC_2_ powder, lithium fluoride (LiF), hydrochloric acid (HCl, 12 M), thioacetamide (TAA), vanadium phosphate sodium (Na_3_V_2_(PO_4_)_3_), ac ethylene black, polyvinylidene difluoride (PVDF), *N*-methylpyrrolidone (NMP), ethanol (AR, 99.5%) are ordered from Aladdin Reagents (Shanghai) Co., Ltd. All chemicals are used directly without any further treatment.

### Preparation of MXene Suspension

One gram of Ti_3_AlC_2_ powder was mixed with 1 g LiF and 20 mL 12 M HCl and stirred at 35 °C for 24 h. The mixture was washed by centrifugation at 3500 r min^−1^ several times until the pH of the supernatant turned to 6 ~ 7. MXene precipitate was collected and mixed with 50 mL of deionized water. Ultrasonic treatment of the mixture was performed under argon for 6 h. A single-layer MXene suspension was then obtained by centrifugation at 3500 r min^−1^ for 1 h.

### Synthesis of FeS_2_ QD/MXene and Fe^2+^/MXene

The 2 mL of FeCl_2_ solution (1 M) was added into 20 mL MXene suspension. After standing for 10 min, Fe^2+^ induced MXene nanosheets to form a three-dimensional network structure. After freeze-drying for 48 h, the 3D MXene-loaded Fe^2+^ composite (Fe^2+^/MXene) was obtained, and the mass ratio of Fe^2+^/MXene is ~ 1.12:1. Then, the TAA and prepared composite with a mass ratio of 5:1 are, respectively, placed into two quartz boats; the Fe^2+^/MXene powders are vulcanized at 300 °C for 2 h in pure Ar_2_ atmosphere (2 °C min^−1^). After naturally cooling down to room temperature, the FeS_2_ QD/MXene is achieved.

In addition, the quantum dot structures with other metal ions were realized by same operation (adding 2 mL MCl_2_ solution, M = Co^2+^, Ni^2+^ into 20 mL MXene suspension).

### Synthesis of FeS_2_ /MXene

MXene suspension (40 mL) was put into a Teflon liner (80 mL) followed by a certain amount of FeCl_2_ solution. After stirring for 30 min, the mixed solution was treated by hydrothermal reaction in a stainless-steel reaction still under 150 °C for 10 h. And the cooled reaction mixture was centrifuged 3–5 times with deionized water and dried by freeze-drying to obtain FeS_2_/MXene.

## Results and Discussion

### Design Principle and Structural Characterizations

**Fig. 1:**
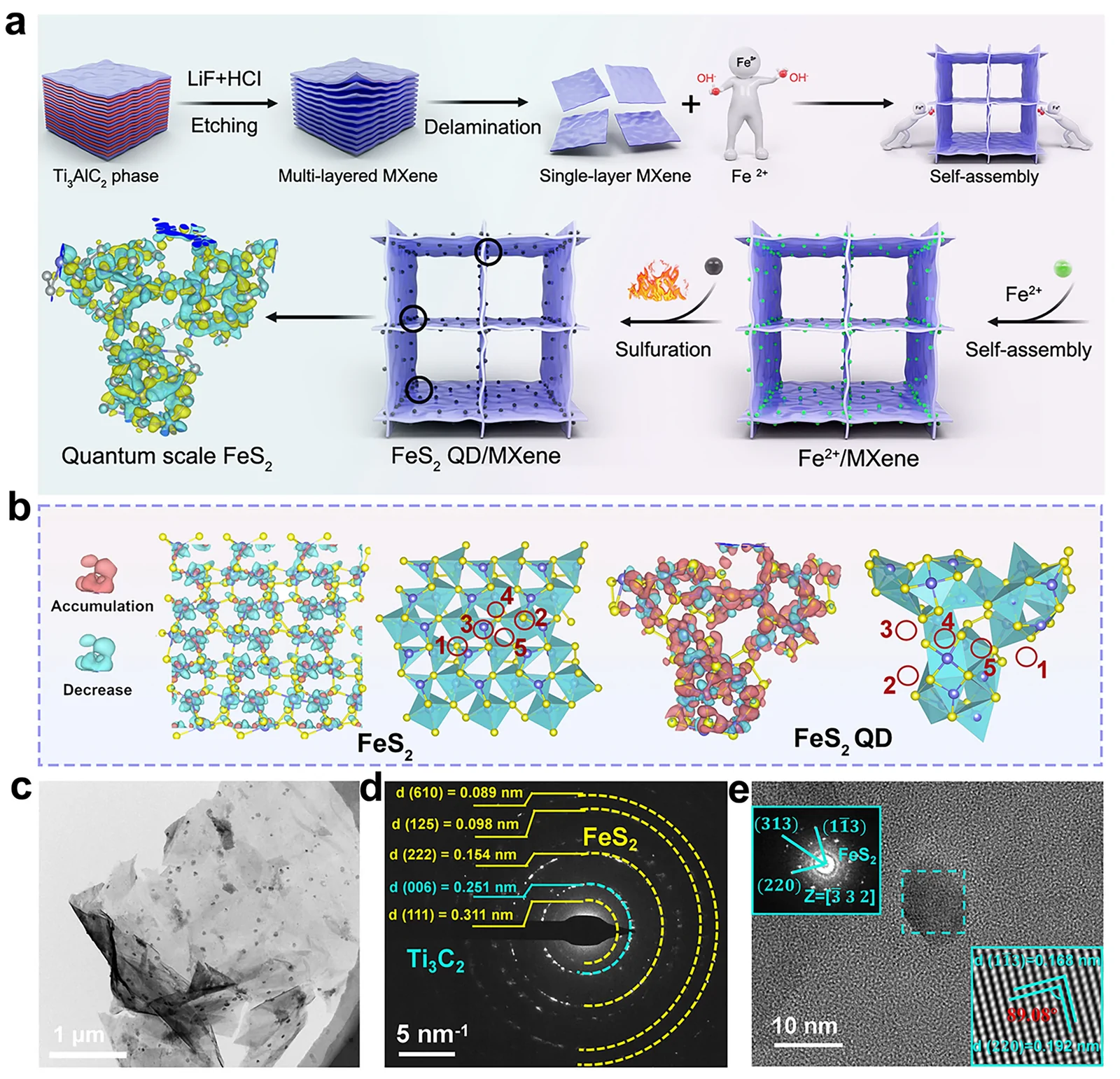
**a** Schematic illustration of synthesis process about FeS_2_ QD/MXene. **b** Schematic representation of the optimization mechanism of the quantum-size effect. **c** TEM image, **d** SAED pattern, **e** HRTEM image of FeS_2_ QD/MXene

Theoretical calculations were conducted to predict the effects of size variations on the local electronic coordination environment and charge transport efficiency of the FeS_2_ structure before the experimental investigation (Fig. 1a, b). The difference charge density (DCD) of FeS_2_ and FeS_2_ QD was calculated based on the prior theoretical assumptions (Fig. 1b), and the depletion regions and charge accumulation were, respectively, depicted in blue and red. Obviously, electron rearrangement could be observed at the crystal boundaries and edges of the FeS_2_ QD structure, which means that abundant unpaired S atoms serve as negative charge centers, facilitating the bonding of metal atoms. Moreover, to assess their sodium storage capacities, we have identified five possible adsorption sites for Na atom on both FeS_2_ and FeS_2_ QD models and calculated the Na adsorption energy using Eq. S1 to assess their sodium storage capacities. As shown in Fig. S1, the weakest adsorption occurs when the Na atom is located above the Na–S bond (site 4); the phenomenon consistently could be similarly observed in the FeS_2_ QD structure (site 5). This can be attributed to the stable Fe–S bonding, where the saturation of surrounding electrons diminishes the capability to bind Na atoms. The vacancy region (site 5) was the most favorable adsorption site, exhibiting the highest adsorption energy (− 2.25 eV), which contributed to the elevated electron density at the vacancy, and facilitate the formation of Na–S bonds. Obviously, the restricted electronic activity of bulk FeS_2_ elevates kinetic barriers for initial Na^+^ insertion, resulting in insufficient capture and accommodation of Na^+^ during the conversion reaction [26]. This is exacerbated by the structural integrity of basal planes, which anchors electrons in symmetric orbitals, limiting active sites for Na^+^ adsorption. Meanwhile, lower electronic conductivity induces severe voltage polarization during cycling, reducing accessible sodium ions and degrading capacity [27]. Consequently, the construction of quantum dot structures played a crucial role in disrupting the coordination integrity of basal planes and forming additional edge free electrons, thus enhancing electrochemical activity. The edge positions in FeS_2_ QD enriched with defects became new preferential adsorption sites (site 1–3), where the calculated adsorption energies reach − 3.73, − 3.92, and − 3.85 eV, significantly exceeding those observed in FeS_2_. The foreign Na^+^ could preferentially bond with the unsaturated edge S atoms of the edge defect, establishing a strengthened interatomic interaction, and demonstrating a stronger chemical affinity and higher adsorption energy on FeS_2_ QD. The above calculation results highlight the fast-ion diffusion and storage capabilities due to the delocalized electronic engineering induced by quantum dot structure, which are expected to deliver excellent wide-temperature performance. The DFT calculations provided critical guidance for the structural design. Specifically, theoretical predictions revealed that FeS_2_ QD exhibit enhanced charge delocalization at edges due to unsaturated sulfur coordination, which directly motivated our strategic focus on synthesizing quantum-sized FeS_2_ to harness these advantages. Consequently, the synthesis strategy of confining FeS_2_ on 3D MXene framework was to stabilize QD. As illustrated in Fig. 1a, the controllable preparation of FeS_2_ QD/MXene composite was obtained. The Ti_3_C_2_ MXene was first obtained by a LiF/HCl etching method, followed by Fe^2+^-induced self-assembly where MXene nanosheets were interconnected through Fe^2+^ ions to construct an interlinked 3D porous substrate, providing an optimal skeleton for FeS_2_ QD anchoring.

A detailed morphology evolution of FeS_2_ QD/MXene was characterized using scanning electron microscope (SEM) and transmission electron microscope (TEM). As displayed in Fig. S2a, the MXene substrate possesses a two-dimensional morphology, but the inherent self-restacking could reduce the electrochemically active surface area and prevent Na^+^ diffusion. The Fe^2+^ ion-induced method could overcome the electrostatic repulsion between the MXene nanosheets and connect them to construct a 3D MXene skeleton (Fig. S2), which suppresses the self-stacking phenomenon and improves the interfacial utilization. The strong interaction between Fe^2+^ and Ti_3_C_2_ surface could guarantee the uniformity distribution of Fe^2+^ species during the subsequent sulfuration treatment, which can inhibit the agglomeration of highly active FeS_2_ QD. The nitrogen adsorption–desorption isotherm curves in Fig. S3 further verified the effective synthesis of porous structures. Distinct from MXene (13.9 m^2^ g^−1^) and FeS_2_/MXene (14.4 m^2^ g^−1^), the Fe^2+^/MXene and FeS_2_ QD/MXene, respectively, showed a higher specific surface area (SSA) of 24.7 and 27.2 m^2^ g^−1^, which are contributed to the mesopores indicated by the type IV adsorption isotherm [28, 29]. Pore size distribution diagram exhibited that the average pore size distribution of FeS_2_ QD/MXene composite is concentrated in 2–6 nm, showing a typical mesoporous feature. The obvious hysteresis ring in curves also proved the existence of mesoporous structures [30]. The increased SSA and porous structure could provide additional transport channels, which can not only facilitate electrolyte infiltration during cycling but also alleviate the volume changes.

TEM images of FeS_2_ QD/MXene described that FeS_2_ QD existed with a diameter of 5–8 nm homogeneously dispersed on the MXene surface (Figs. 1c and S4), which maintain uniform dispersion characteristics. In Fig. 1d, the selected area electron diffraction (SAED) pattern image clearly exhibited diffraction rings assigned to the Ti_3_C_2_ and FeS_2_, which, respectively, coincide with the (006) crystal plane of Ti_3_C_2_ and the (111), (222), (125), and (610) planes of FeS_2_ [31, 32]. The clear lattice shown in Fig. 1e indicated good crystallinity of FeS_2_ QD, and the distance of 0.168 nm and 0.192 nm could, respectively, be ascribed to ($\overline{1 }$ 13) and (220) lattice plane. In addition, the high-angle annular dark-field (HAADF) image and elemental mapping analysis of FeS_2_ QD/MXene composite could confirm the presence and homogeneous distribution of Fe, S, Ti, C, and O species, certifying the uniform dispersion of the FeS_2_ particle on the Ti_3_C_2_ MXene (Fig. S5). Moreover, the thermogravimetric analysis (TGA) was applied to estimate the weight ratio of FeS_2_ in FeS_2_ QD/MXene, and the calculated ratio was around 36.6% (Fig. S6).

### Interaction Characterization and Mechanical Properties

**Fig. 2:**
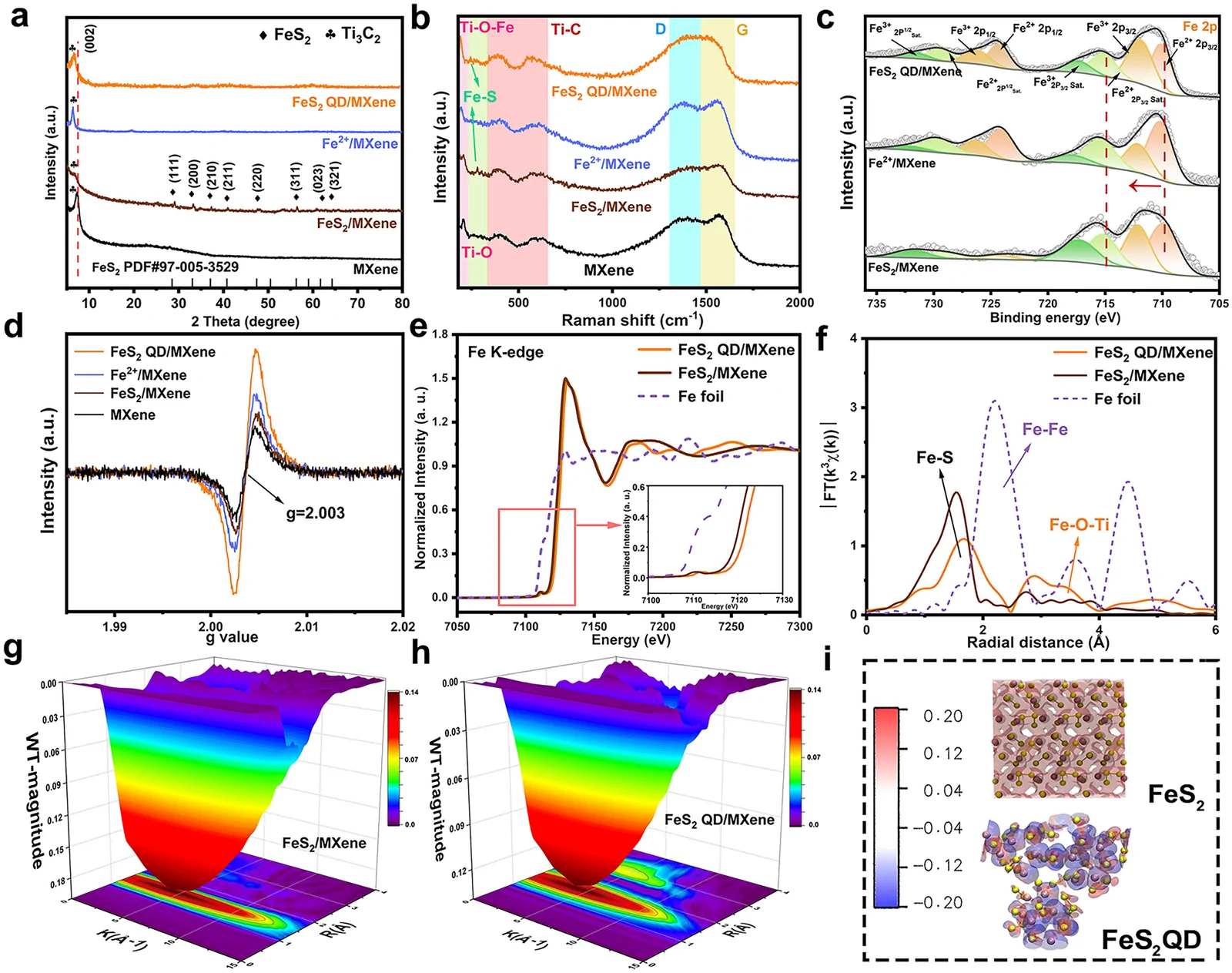
**a** XRD patterns and **b** Raman spectra, **c** high-resolution XPS spectra of Fe 2*p* and **d** electron paramagnetic resonance images of MXene, FeS_2_/MXene, Fe^2+^/MXene and FeS_2_ QD/MXene. **e** Normalized K-edge XANES and **f** FT-EXAFS in R space for FeS_2_/MXene, FeS_2_ QD/MXene and Fe foil. **g–h** Wavelet transforms for the k^3^-weighted EXAFS signals of FeS_2_/MXene and FeS_2_ QD/MXene. **i** Electrostatic potential diagrams of FeS_2_ and FeS_2_ QD

X-ray diffraction (XRD) patterns are shown in Fig. 2a; the broadened (002) peak could be observed in MXene, FeS_2_/MXene, Fe^2+^/MXene and FeS_2_ QD/MXene composites attributing to Ti_3_C_2_ substrate. In addition, the (002) peak of Fe^2+^/MXene and FeS_2_ QD/MXene shifted to the lower angel, which proves that metal ion gelation method effectively promotes the expansion of layer spacing and the construction of three-dimensional structures [28]. Diffraction peaks of FeS_2_ QD/MXene match well with planes of FeS_2_ (JPDS No. 97-5-3529) in FeS_2_/MXene composite with slightly reduced intensity, which might be attributed to the defect-rich structure. Raman spectra were analyzed to identify chemical bonding states and molecular structures evolution (Figs. 2b and S7). Peaks located at around 271 cm^−1^ were attributed to the Fe–S bands of FeS_2_ in FeS_2_ QD/MXene and FeS_2_/MXene, while the peaks located at around 420 and 620 cm^−1^ are, respectively, corresponding to the vibrations of Ti–C bonds of Mxene [33, 34]. And the bands between 1250 and 1750 cm^−1^ were, respectively, corresponding to the D-band and G-band of Ti_3_C_2_ Mxene [35, 36]. It is obvious that peaks belonging to Ti–C and Fe–S bonds in the FeS_2_ QD/MXene composite shifted toward the low wavenumbers than that of FeS_2_/MXene. This phenomenon could be attributed to the formation of Ti–O–Fe bonds located at 190 cm^−1^, the presence of which inevitably promotes charge rearrangement around the active sites, resulting in electron density decreasing near the Fe–S and Ti–C bonds, and causing a redshift of the corresponding peaks [37]. In addition, the Ti–O–Fe peaks of FeS_2_ QD/MXene showed relatively higher strengths compared with Fe^2+^/MXene and FeS_2_/MXene counterparts, indicating the excellent interfacial coupling. A series of analyses of chemically bonded coordination environments and states proved that quantum-sized FeS_2_ triggered charge distribution change and electron diffusion, which could be beneficial for the ion storage performance.

X-ray photoelectron spectroscopy (XPS) measurements were taken to analyze the electronic structure of prepared materials. The spectra, respectively, attributed to the Ti–C 2*p*_3/2_, Ti–C 2*p*_1/2_, Ti(II) 2*p*_3/2_, Ti(II) 2*p*_1/2_, Ti(IV) 2*p*_3/2_ and Ti(IV) 2*p*_1/2_, depicted in Fig. S8a [38]. The Ti 2*p* peak positions of FeS_2_ QD/MXene showed a positive shift compared to FeS_2_/MXene, indicating a decrease in the electron density. As for the Fe elements (Fig. 2c), it could be deconvoluted into eight peaks, respectively, assigned to Fe(II) 2*p*_3/2_, Fe(II) 2*p*_1/2_, Fe(III) 2*p*_3/2_ and Fe(III) 2*p*_1/2_ [39, 40]. Notably, the Fe 2*p* and S 2*p* peaks of the FeS_2_ QD/MXene and Fe^2+^/MXene samples were shifted toward higher binding energies compared to these of FeS_2_/MXene, indicating that the electronic structure is obviously different from that of FeS_2_/MXene due to the abundant defects. The coordination environment and bonding state proved that the quantum size triggered charge delocalization, thus maximizing the concentration of uncoordinated electrons at FeS_2_ edge, which could optimize the sodium storage and transport ability. As depicted in Fig. 2d, the electron paramagnetic resonance (EPR) measurements illustrated that charge rearrangement could produce abundant structural defects. The presence of unpaired electrons caused four samples to exhibit a symmetric Lorentzian line with a g value of around 2.003 [41]. Moreover, the FeS_2_ QD/MXene composite possessed the highest vibration intensity, indicating that the largest defect concentration generated in crystal structure. The phenomenon revealed that highly dispersed FeS_2_ QDs could deliver a mass of free unpaired electrons, which could regulate the charge distribution and optimize the phase disorder and further increase the Na^+^ ions storage sites exposure and shorten ion-transfer distance to achieve significant wide-temperature reversible capacity.

To accurately analyze the local coordination environment of Fe atoms, X-ray absorption spectroscopy (XAS) was performed. The Fe K-edge XAFS (Fig. 2e) showed that the Fe absorption edge for FeS_2_ QD/MXene shifts toward higher energy than that of FeS_2_ QD/MXene, further indicating the more positively charged state of central Fe atoms, which is consistent with XPS results. It demonstrated that efficient electron transfers from Ti_3_C_2_ interface to FeS_2_ QD, leading to the charge delocalization around active sites [42, 43]. As depicted in the K-edge FT-EXAFS in R space (Fig. 2f), the white-line peak intensity of FeS_2_ QD/MXene is smaller than that of FeS_2_/MXene, which means that charge rearrangement induced by quantum size leads to enhanced electron density in Fe 3*d* orbitals [44]. Furthermore, the coordination distance of FeS_2_ QD/MXene (1.65 Å) contributed to Fe–S path significantly larger than that of FeS_2_/MXene, and the enhanced bond length implied the optimized delocalized electron regions. As demonstrated in fitting results (Table S1), the FeS_2_ QD/MXene curve shows a distinct peak at 3.41 Å attributed to Fe–O–Ti coordination path, demonstrating the strong interfacial coupling between FeS_2_ and Ti_3_C_2_ caused by quantum-size effect [45]. For FeS_2_ QD/MXene, the coordination numbers of the Fe–S and Fe–O–Ti paths are, respectively, 5.25, 1.41, and 2.09, which are smaller than those of FeS_2_/MXene (6.56 and 2.44), demonstrating the more unsaturated coordination construction in quantum dot structure. The wavelet-transformed (WT) contour plots for Fe K-edge are shown in Figs. 2g–h and S9. The maximum intensity of FeS_2_ QD/MXene at 4.05 Å^−1^ is assigned to the Fe–S coordination paths, which is closer to that of FeS_2_/MXene, indicating the preserved FeS_2_ lattice structure. In addition, another weaker intensity relative maximum at 3.92 Å^−1^ corresponding to appears for FeS_2_ QD/MXene. It is evident that quantum-size-induced edge defects could change the coordination state surrounding Fe atoms, thereby inducing charge delocalization in active sites. The electronic structure optimization is beneficial to promote the multi-electron transition reaction, thus conferring FeS_2_ QD/MXene wide-temperature performance superiority.

To further exposure the relationship between electronic structure/properties and electrochemical properties based at atomic scale, the electrostatic potentials, energy band structures and d-band centers have been investigated based on DFT calculations. The optimized model of FeS_2_ is constructed in Fig. S10a, and the quantum dot structure was successfully constructed by maximizing the defect concentration at the edge of the FeS_2_ lattice (Fig. S10b). In anode materials, when the electronic structure of electrodes could act as a prominent electron donor for coordination with Na^+^ ions, the distinctive donor feature was the high negative surface electrostatic potential [46, 47]. As shown in Fig. 2i, the electrostatic potential (ESP) pictures demonstrated the quantum-size effect, and the negative potential was mainly localized around the sulfur atoms in FeS_2_ structure. While in FeS_2_ QD structure, edge atoms possessed a high number of uncoordinated electrons, preventing the electron conjugated orbital overlap, and results in more negative ESP than that of the intact crystal structure. Hence, the FeS_2_ QD structure was more likely to combine with Na^+^ and participate in electrochemical reactions. Excellent crystal edge carrier transfer caused electron rearrangement and energy level shift of FeS_2_ QD, which plays a vital role in optimizing band structure and regulating band gaps. As a result, FeS_2_ QD exhibited a lower band gap value (0.02 eV) compared to integrated FeS_2_ structure (0.52 eV) (Fig. S11). Moreover, it can be observed in Fig. S12 that the d-band center (*ε*_d_) of Fe atoms in FeS_2_ QD moved from − 2.47 to − 1.20 eV, which was much closer to the Fermi level and contributed to reduce the anti-bonding orbital filling of delocalized electrons, and increase the adsorption strength between active center and Na atoms [48]. In conclusion, the characterization tests and theoretical calculations conjointly proved that the high-concentration unsaturated coordination environment induced by quantum size could enrich electrochemical active centers, providing a possibility for optimizing electrochemical properties and ion diffusion kinetics under wide temperature.

**Fig. 3:**
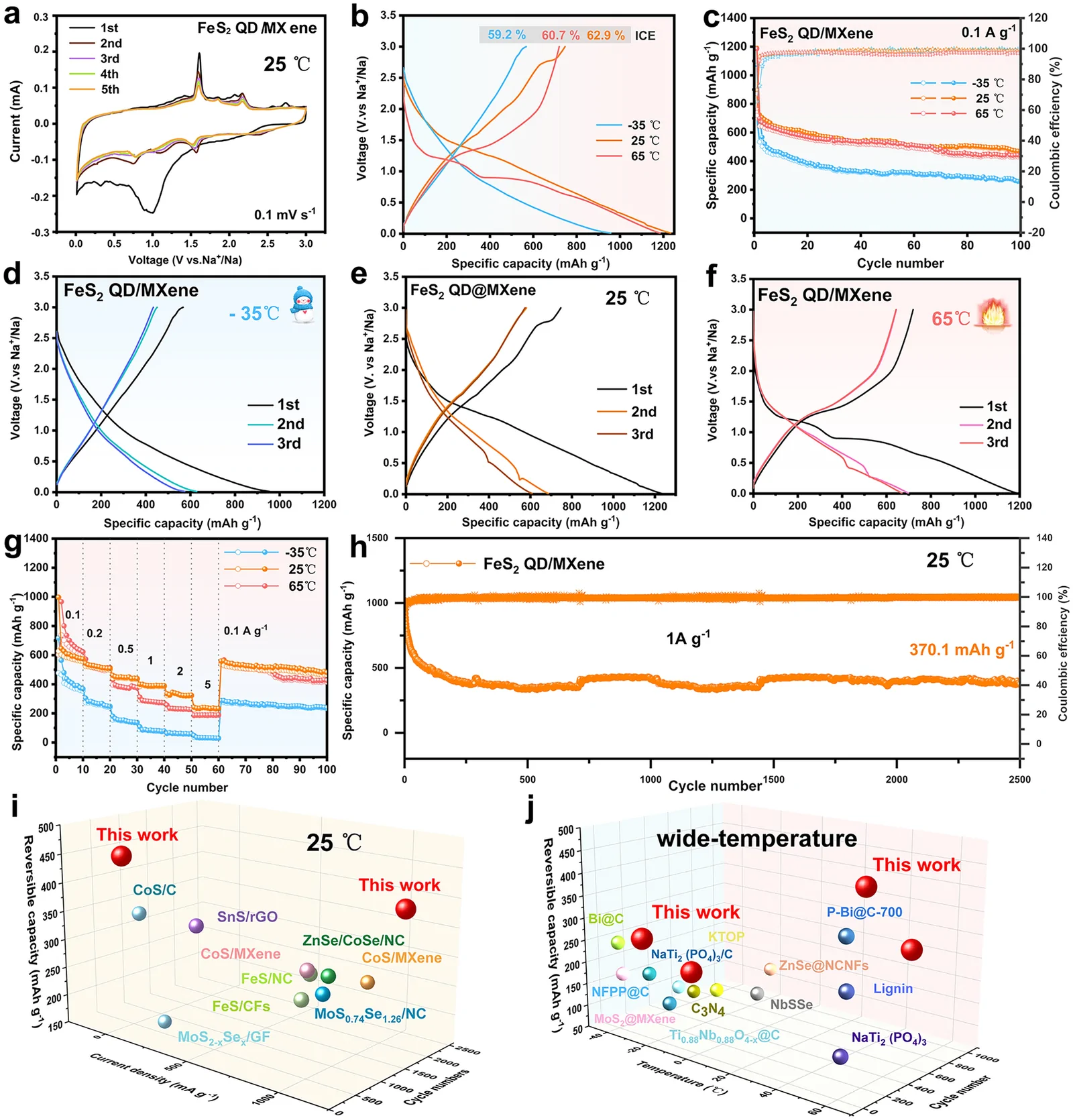
**a** CV curves at 0.1 mV s^−1^ in 0.01–3.0 V of FeS_2_ QD/MXene at 25 °C. **b** GCD curves at first cycle and **c** cycle performance at 0.1 A g^−1^ at different temperatures. GCD profiles for first five cycles at 0.1 A g^−1^ at **d** − 35 °C, **e** 25 °C and **f** 65 °C. **g** Rate capability at increasing current densities at different temperatures. **h** Cycling performance at 1.0 A g^−1^. **i** Comparison of performance of FeS_2_ QD/MXene with the reported TMDs-based anode for SIBs at room temperature and **j** wide temperature

To investigate the collaborative optimization mechanism of quantum-size effect and 3D porous structure on Na^+^ storage and transport kinetics, the electrochemical behavior based on prepared MXene, FeS_2_/MXene, Fe^2+^/MXene and FeS_2_ QD/MXene anodes was examined in sodium-ion half-cells. As shown in Fig. 3a, the cyclic voltammetry (CV) tests illustrated that a broad reduction peaked at 1.0 V, disappearing in subsequent cycles assigned to the formation of solid electrolyte interface layer (SEI) during the initial Na^+^ insertion in FeS_2_ QD/MXene anode [49]. Meanwhile, other reduction peaks located at 1.54, 1.14 and 0.76 V, respectively, ascribed to the intercalation of Na^+^ into FeS_2_ and the subsequent conversion of Na_x_FeS_2_ into Fe and Na_2_S [50]. In the following anodic sweeps, the peak at around 1.58 V could be related to the desodiation of Na_x_FeS_2_, and oxidation peaks at around 2.17 and 2.7 V might be associated with transformation of FeS_2_. What’s more, the subsequent CV profiles of the FeS_2_ QD/MXene electrode were well overlapped, reflecting its excellent cycle performance and outstanding cycling stability [51].

FeS_2_ QD/MXene anode, respectively, delivered initial coulombic efficiency (ICE) of 62.9%, 60.7% and 59.2% at 25, − 35 and 65 °C, which is established in Fig. 3b. The lower ICE is contributed to the electrolyte being more easily decomposed resulting in more side reactions at low or high temperature [52]. As shown in Fig. S13a, the FeS_2_ QD/MXene anode possessed the greater ICE and discharged specific capacity in comparison with the MXene (22.6%), FeS_2_/MXene (23.7%) and Fe^2+^/MXene (40.1%) electrodes, demonstrating that the high defect concentration and multiple active centers induced by quantum dot structure possess superior electrochemical reaction activity [53]. Furthermore, the GCD curves overlap well in subsequent cycles, which confirm the excellent stability of FeS_2_ QD/MXene electrode for sodium-ion storage. Moreover, the cycling performance of FeS_2_ QD/MXene anode at a current density of 0.1 A g^−1^ with the voltage range of 0.01–3.0 V for SIBs at different temperatures is shown in Fig. 3c. After 100 cycles, the well-designed FeS_2_ QD/MXene anode could, respectively, deliver high capacities of 463.8, 255.2 and 424.9 mAh g^−1^ under 25, − 35 and 65 °C. On the contrary, an apparent irreversible capacity fading observed in FeS_2_/MXene curve (Fig. S13b), which could be attributed to the large FeS_2_ particles, would undergo volume expansion during the charge–discharge process, making them prone to detachment from the current collector, and leading to rapid capacity degradation. The MXene and FeS_2_ electrodes could, respectively, possess a low specific capacity of 77.2 mAh g^−1^ (Fig. S13) and 58.9 mAh g^−1^ (Fig. S14) at 0.1 A g^−1^ after 100 cycles. But the FeS_2_ QD/MXene electrode could deliver a significantly increased capacity of 463.8 mAh g^−1^ at the same current density, which is superior to either single component. Apparently, the improved wide-temperature electrochemical performance was mainly benefiting from the synergistic influence of the three-dimensional hierarchical structure and quantum-size effect. The first three galvanostatic charge/discharge (GCD) profiles of FeS_2_ QD/MXene at 0.1 A g^−1^ under wide temperatures are shown in Fig. 3d–f. The first charge capacity at − 35 °C (951.4 mAh g^−1^) was lower than the capacity of 1200.2 mAh g^−1^ obtained at 25 °C, which was mainly attributed to the cold service temperature tending to enhance Na^+^ ions diffusion barrier, leading to significant capacity fading [54]. Moreover, control experiments under identical low-temperature electrolyte (1 M NaClO_4_/EC:PC) at − 35 °C are illustrated in Fig. S15; the MXene, FeS_2_ QD/MXene and Fe^2+^/MXene electrodes, respectively, deliver capacities of 20.8, 30.3 and 33.4 mAh g^−1^ after 100 cycles at 0.1 A g^−1^, which is considerably lower than FeS_2_ QD/MXene (255.2 mAh g^−1^), suggesting that quantum-size effects rather than electrolyte variations drove the improved wide-temperature performance.

The optimized Na^+^ transport kinetics in FeS_2_ QD/MXene composite were demonstrated by electrochemical impedance spectroscopy (EIS) [55]. As shown in Fig. S16a, the fitting* R*_ct_ value of FeS_2_ QD/MXene (193.0 Ω) was smaller than that of MXene (866.7 Ω), FeS_2_/MXene (734.1 Ω) and Fe^2+^/MXene (628.5 Ω) anodes, indicating the faster ion/electron transfer of abundant edge defects regions. The relationship between impedance and the phase angle is shown in Fig. S16b, and the FeS_2_ QD/MXene electrode exhibited the smallest Warburg factor (*σ*), indicating the fastest Na^+^ diffusion efficiency shown in Table S2. The calculated diffusion coefficient of Na^+^ ion (D_Na_^+^, cm^2^ S^−1^) in FeS_2_ QD/MXene anode was 1.17 × 10^–8^ cm^2^ S^−1^, which is higher than that of MXene (6.60 × 10^–9^ cm^2^ S^−1^), FeS_2_/MXene (4.72 × 10^–9^ cm^2^ S^−1^) and Fe^2+^/MXene (2.95 × 10^–9^ cm^2^ S^−1^) electrodes, suggesting that the ultra-fine quantum dot structure could contribute to shorter ions transport distances and enhanced electrolyte penetration for superior charge transfer [56]. Hence, the quantum-sized FeS_2_ tended to display significantly rate performance (Fig. 3g), when the current density returns to 0.1 A g^−1^, the FeS_2_ QD/MXene anode still recovered back to deliver excellent capacities of 235.3, 464.3 and 426.1 mAh g^−1^ under − 35/25/65 °C, respectively. The high capacity that achieved adequately reveals prominent fast-charging capability of the FeS_2_ QD/MXene anode under wide ambient temperature. And the capacity decay at 65 °C is relatively faster than that at 25 °C, which is due to the instability of the electrolyte at high temperatures.

As shown in Fig. 3h, based on full active sites utilization and fast surface interfacial ion reaction kinetics, the FeS_2_ QD/MXene anode exhibited the best long-cycle performance (370.1 mAh g^−1^) after 2500 cycles at the high current density of 1 A g^−1^. In contrast, MXene, Fe^2+^/MXene and FeS_2_/MXene electrodes have poor long-term cycling stability, with capacity, respectively, decreasing to 8.6, 33.4 and 132.7 mAh g^−1^ after 1600, 1700 and 1900 cycles (Fig. S17). Obviously, the high concentration of edge uncoordinated electrons induced by quantum-size FeS_2_ excites electrochemical activity, attributed to the electrochemical properties of well-designed and also possess superior cycling properties than previous TMD-based electrodes compared with other techniques [S14–S21] (Fig. 3i and Table S3). Besides, the FeS_2_ QD/MXene electrode also delivers excellent long-term cycling performance under the wide operation temperatures. As shown in Figs. S18 and S19, after 500 cycles at 0.5 A g^−1^, the anode, respectively, obtained capacities of 121.5 and 312.5 mAh g^−1^at -35 and 65 °C. Compared to previously reported works, FeS_2_ QD/MXene electrode constructed based on quantum dot effects and 3D MXene skeleton exhibits significant advantages under wide temperature [S22–S33] (Fig. 3j and Table S4).

### Mechanism Investigation

**Fig. 4:**
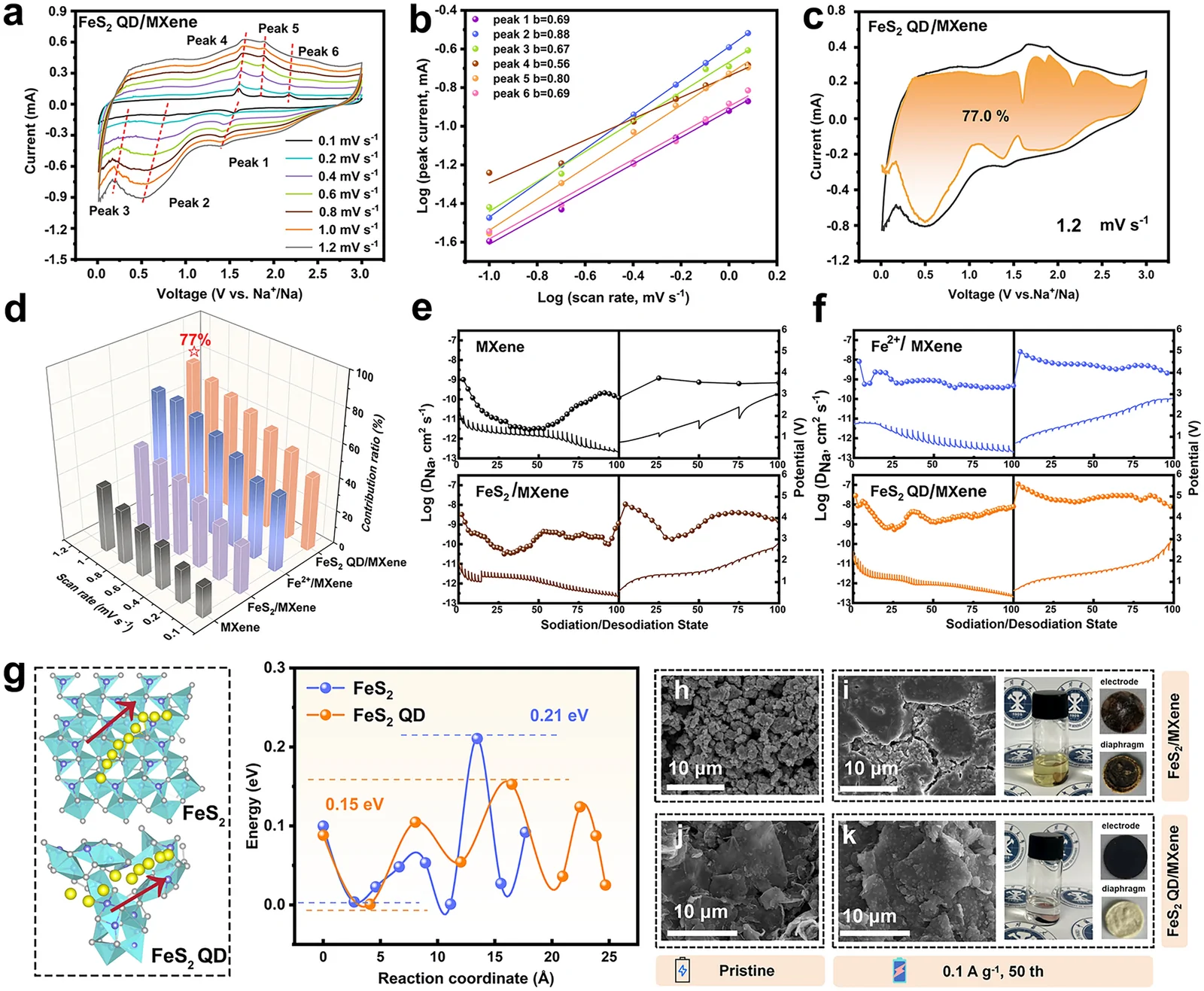
**a** CV curves at different scan rates. **b** Linear relationship of peak currents versus scan rates. **c** Capacitive contribution curve for FeS_2_ QD/MXene anode at 1.0 mV s^−1^. **d** Capacitive contribution ratios and **e–f** GITT potential profiles and Na^+^ ions diffusion system of electrodes. **g** Na^+^ ions diffusion routes across and the corresponding diffusion energies of FeS_2_ and FeS_2_ QD/MXene electrodes. **h–k** SEM images, post cycled electrodes/separators photographs after cycling of FeS_2_/MXene and FeS_2_ QD/MXene anodes

Electrochemical testing has convincingly demonstrated that the integration of unsaturated coordination active sites with regulated heterogeneous structures regulation can synergistically enhance Na^+^ storage properties of FeS_2_ QD/MXene electrode. To delve deeper into the kinetic mechanism of the prepared FeS_2_ QD/MXene anode in SIBs, CV tests are conducted at varying scan rates from 0.1 to 1.2 mV s^−1^. As illustrated in Fig. 4a, the peak positions and shapes of the CV profiles were consistently preserved across progressively increasing scan rates. The relationship between peak current (*i*) and scan rate (*v*) adheres to the following empirical formulas [57]:

1 $$i=a{v}^{b}$$

2 $$\mathrm{log}i=\mathrm{blog}v+\mathrm{log}a$$

where the* b* approaches 1, the electrochemical reaction is predominantly governed by pseudocapacitance, as well as *b*-value of 0.5 signifies a purely diffusion-dominated process. The calculated* b*-values of FeS_2_ QD/MXene electrode are shown in Fig. 4b. Compared with the Fe^2+^/MXene (Fig. S20), FeS_2_/MXene (Fig. S21) and MXene (Fig. S22) anodes, the FeS_2_ QD/MXene electrode was mainly based on the highly active hetero-interface-induced capacitance process control for Na^+^ ions storage, which exhibits enhanced charge transport characteristics. In addition, the capacitive contribution ratio can be estimated according to following equations [58]:

3 $$i ={ k}_{1}v+{ k}_{2}{v}^\frac{1}{2}$$

4 $$\frac{i}{{v}^\frac{1}{2}}={k}_{2}{v}^\frac{1}{2}+{k}_{1}$$

in which ${k}_{1}v$ and ${k}_{2}{v}^\frac{1}{2},$ respectively, stand for the pseudocapacitive process and diffusion-dominated processes. As depicted in Fig. 4c, d, it can be concluded that the contribution of the pseudocapacitance-controlled behavior increases as the scan rate increases from 0.1 to 1.2 mV s^−1^. When the scan rate achieves 1.2 mV s^−1^, the dominating capacitive contribution of FeS_2_ QD/MXene electrode depicted a value of 77.0%, significantly surpassing those of the Fe^2+^/MXene (68.9% at 1.2 mV s^−1^), FeS_2_/MXene (51.9% at 1.0 mV s^−1^) and MXene (39.2% at 1.0 mV s^−1^) anodes. The high pseudocapacitance contribution could be related to the uncoordinated active sites and fast-ion diffusion transport, which due to the high-concentration edge defects, strong interfacial coupling and highly efficient charge transport of FeS_2_ QD/MXene composites, contributing to excellent electrochemical reaction kinetics. Hence, the FeS_2_ QD/MXene anode showed much higher D_Na_^+^ kinetics than other electrodes (Figs. 4e, f and S23). The FeS_2_ QD/MXene electrode also exhibits fast Na^+^ ions transport over wide-temperature range, the GITT curves are shown in Figs. S24 and S25. In particular, the D_Na_^+^ at − 35 °C is only two orders of magnitude lower than that of room temperature, which further confirming that unsaturated coordinated edge atoms of FeS_2_ QDs could significantly enhance the Na^+^ diffusion behaviors. Moreover, DFT calculations were also conducted to explore the sodium migration phenomenon, with the optimized migration paths exhibited in Fig. 4g. Compared with the integrated FeS_2_ lattice structure, the FeS_2_ QD structure featured a higher concentration of edge uncoordinated atoms and grain boundary active sites. The migration paths indicated that Na atoms navigating through the FeS_2_ QD structure were less restricted by the intrinsic lattice constraints, thus exhibiting greater diffusion freedom and delocalization possibilities. This structural advantage likely facilitates the formation of Na_2_S at defect-rich edge sites, resulting in lower diffusion barriers (0.15 eV) and promoting low-impedance Na^+^ migration [59].

To further elucidate this reaction process, cells after cycling were disassembled to demonstrate electrode morphology and diaphragm state. In Figs. 4h-k and S26, the MXene, FeS_2_/MXene, Fe^2+^ MXene anodes exhibited obvious agglomeration and fragmentation after charging/discharging process with severe electrode splitting. In comparison, the FeS_2_ QD/MXene anode maintained structural integrity after 50 cycles at 0.1 A g^−1^, accounting for its exceedingly electrochemical performance and long-cycle stability. Additionally, cross-sectional SEM images revealed that the FeS_2_/MXene anode is dislodged from the fluid collector and the cross-sectional thickness expansion of up to 52.1% (Fig. S27), compared to only 16.2% for the FeS_2_ QD/MXene electrode (Fig. S28). As depicted in Fig. 4h, i, when recycled electrodes were immersed in the fresh electrolyte, the FeS_2_/MXene anode presented a cloudy appearance, indicative of a significant shuttle effect. In contrast, distinct yellow deposits were observed on the glass fiber separator of FeS_2_/MXene after cycling. On the contrary, the electrolyte containing FeS_2_ QD/MXene anode remained transparent suggesting effective inhibition of the polysulfide shuttle effect (Fig. 4j, k). The innovative design of the FeS_2_ QD/MXene electrode, featuring unsaturated coordination structures, likely induces local electron delocalization, thereby enhancing charge conversion capabilities and inhibiting the formation of polysulfides and by-products.

**Fig. 5:**
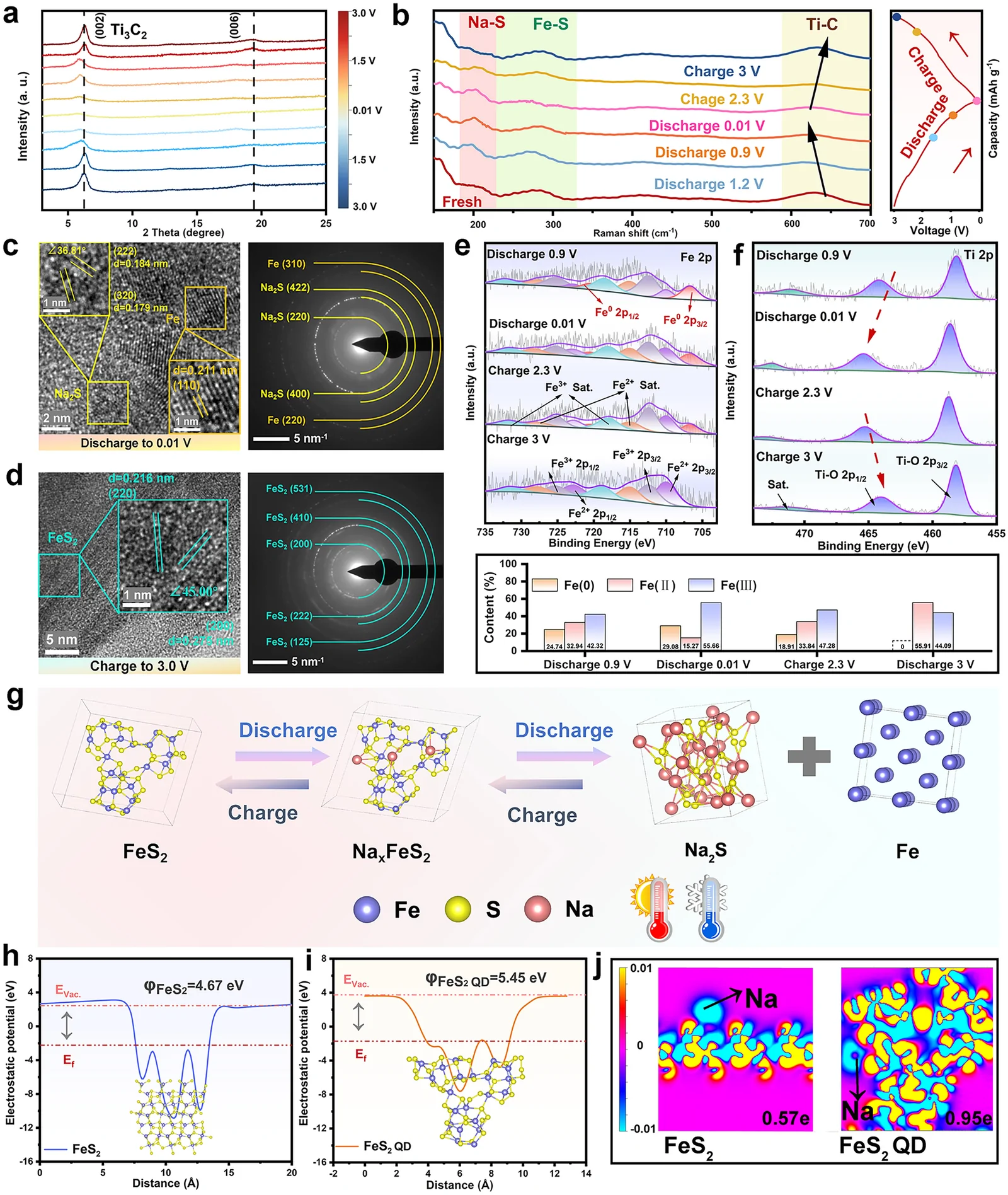
**a** Ex situ XRD, **b** ex situ Raman spectra, **c-d** ex situ TEM images and **e–f** ex situ XPS of FeS_2_ QD/MXene electrodes at different discharge/charge voltages at first cycle. **g** Schematic illustration of sodium storage mechanism based on FeS_2_ QD/MXene electrode. **h–i** Work functions and **j** 2D charge difference plots of FeS_2_ and FeS_2_ QD structures

To comprehensively examine the sodium storage and transport mechanisms of FeS_2_ QD/MXene composite, the ex situ XRD patterns during the first cycle of charging/discharging cycle are shown in Fig. 5a. The distinct reflections from the Ti_3_C_2_ material were observed at 6.2° and 19.4°, corresponding to the (002) and (006) planes, respectively, in the initial state of discharge. As the discharge progressed, the positions of these diffraction peaks gradually shifted toward smaller angles, with a concurrent reduction in intensity, indicating the insertion of Na^+^ ions into the interlayers of the MXene substrate. Notably, upon recharging the electrode to 3.0 V, these peaks reverted to their original positions, demonstrating a highly reversible conversion reaction. Further insights into the conversion reaction mechanism of the FeS_2_ QD/MXene anode were sought through ex situ Raman spectroscopy (Fig. 5b). During the discharge proceeding, the peaks emerging at around 218.3 cm^−1^ gradually appear, which could be attributed to the formation of Na_2_S [60]. Notably, at a discharge voltage of 0.01 V, the peak associated with the Na–S bond reached maximum intensity, whereas the Fe–S peak at approximately 280 cm^−1^ nearly vanished, indicating rapid charge transport within the FeS_2_ QD. This charge interaction appears to facilitate the coordination of Na^+^ ions, enhancing the conversion reaction that produces Na_2_S. Upon charging the electrode to 3.0 V, the peak indicative of the Na–S bond disappeared, and the Fe–S bond peak reappeared, suggesting the reversible regeneration of the active FeS_2_ material. Additionally, peaks at 631.0 cm^−1^, attributed to Ti–C bonds, were observed to red shift to 612.2 cm^−1^ during the discharge process, confirming the successful embedding of Na^+^ ions and the consequent expansion of the MXene interlayer spacing [61]. Crucially, during the subsequent charging process, the peak position fully reverts to its original value of 631.0 cm^−1^, demonstrating Na^+^ deintercalation and confirming the highly reversible nature of the structural changes.

To examine the morphological and microstructural changes during the sodiation/desodiation processes of the FeS_2_ QD/MXene anode, ex situ TEM was employed. Upon discharging the electrode to 0.01 V, HRTEM images revealed the emergence of lattice fringes corresponding to the Na_2_S crystal planes of (222) and (320) (Fig. 5c). Additionally, lattice fringes associated with the (110) plane of metallic Fe were also observed. Importantly, the formation of Fe and Na_2_S hetero-interfaces during discharge appears to facilitate space charge storage at these interfaces, enhancing localized charge redistribution. This enhancement contributes to interfacial pseudocapacitance and stabilizes the reaction kinetics [62]. Furthermore, SAED analysis displayed distinct diffraction rings for the (310) and (220) planes of Fe, as well as the (442), (220), and (400) planes of Na_2_S, consistent with the ex situ XRD patterns. Conversely, upon charging the electrode to 3.0 V, the lattice fringes corresponding to Fe and Na_2_S disappeared (Fig. 5d), with those corresponding to the (220) and (200) crystal planes of FeS_2_ reemerging, indicating excellent reversibility of the composite electrode. This reversible disappearance of hetero-interfaces further confirms the dynamic nature of space charge storage, synergizing with electrode conversion reactions to achieve high reversibility [63, 64]. This dual mechanism underscores the structural and electrochemical benefits of the FeS_2_ QD/MXene architecture.

Additionally, ex situ XPS was conducted to analyze the states of the FeS_2_ QD/MXene electrode at various charging and discharging stages. As shown in Fig. 5e, during the Na^+^ insertion process, peaks at 706.1 and 720.5 eV attributed to the Fe element of the discharge product were consistently present, reaching their maximum intensity (29.08%) at a potential of 0.01 V. The disappearance of these Fe element peaks and the elevated level of Fe (II) after charging to 3.0 V suggests the re-formation of FeS_2_. Moreover, the emergence of peaks attributed to Fe(III) 2*p*_3/2_ and Fe(III) 2*p*_1/2_ indicates the formation of less active Fe(III) oxides on the electrode surface, likely due to irreversible electrolyte side reactions during the initial charge/discharge cycle. Peaks at 458.12 and 464.07 eV, respectively, corresponding to the Ti–O 2*p*_3/2_ and Ti–O 2*p*_3/1_ orbitals were identified, linked to the Ti atom in the FeS_2_ QD/MXene electrode (Fig. 5f). When discharged to 0.01 V, these two peaks shifted to 458.33 and 464.30 eV, suggesting that the Ti–O functional groups had captured Na^+^ ions during sodiation process [65]. When recharged to 3.0 V, the peaks associated with the Ti–O bond returned to lower binding energies, confirming the excellent reversibility of the FeS_2_ QD/MXene electrode. The above discussions systematically clarify the Na^+^ electrochemical reaction mechanism in the FeS_2_ QD/MXene, as depicted in Fig. 5g, and the transformation reaction equations are summarized as follows:

5 $${\mathrm{FeS}}_{{2}} + \, x{\mathrm{Na}}^{ + } + \, x{\mathrm{e}}^{ - } \leftrightarrow {\mathrm{Na}}_{x} {\mathrm{FeS}}_{{2}}$$

6 $${\mathrm{Na}}_{x} {\mathrm{FeS}}_{{2}} + \, \left( {{4} - x} \right){\mathrm{Na}}^{ + } + \, x{\mathrm{e}}^{ - } \leftrightarrow {\text{ 2Na}}_{{2}} {\text{S }} + {\text{ Fe}}$$

First-principles calculations were performed to elucidate the mechanisms underlying the highly reversible conversion reaction of the FeS_2_ QD/MXene electrode. The work function results presented in Fig. 5h, i indicate a greater propensity for electron transfer from the integrated FeS_2_ and FeS_2_ QD to the adsorption surface. Compared to FeS_2_ crystals, which have a work function of 4.67 eV, the FeS_2_ QD exhibits an increased work function of 5.32 eV. This increase demonstrates that the quantum-sized structure disrupts the electrochemical inertia of the basal plane atoms, facilitating the transfer of internal electrons to the surface of the FeS_2_ QD [66]. The quantum-size effect modifies the electronic energy and optimizes the surface charge distribution, thus lowering the Na^+^ diffusion energy barrier. This aligns with the observed low *R*_ct_ and high rate performance in electrochemical tests conducted at various temperatures. Additionally, 2D differential charge density calculations quantify the localized electron enrichment phenomenon at the FeS_2_ QD/MXene electrode. As depicted in Fig. 5j, the Bader charge transfer between the FeS_2_ QD and Na atoms reaches 0.95 e, significantly exceeding the 0.57 e of bulk FeS_2_. These findings confirm that charge delocalization and high-concentration defects, driven by the quantum-size effect, contribute to significant improvements in cycling performance and rapid ion diffusion across wide temperature.

**Fig. 6:**
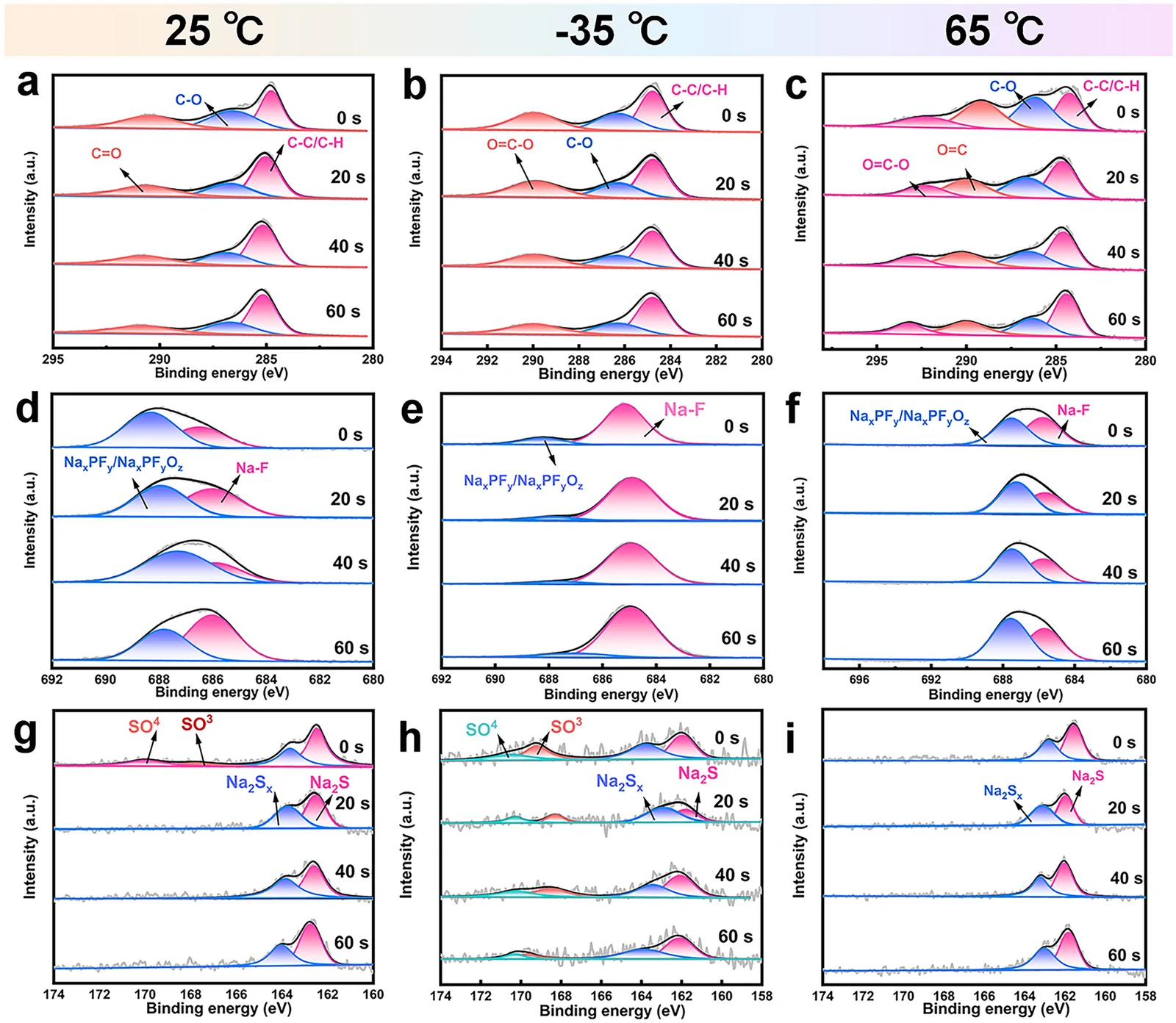
In-depth **a–c** C 1*s*, **d–f** F 1*s* and **g–i** S 2*p* of FeS_2_ QD/MXene electrode after 10 cycles with the sputter time of 0, 20, 40, and 60 s under 25, − 35 and 65 °C

Moreover, to investigate the composition and vertical distribution of the SEI after cycling at different temperatures, XPS depth profiling on anodes after 10 cycles was performed (Fig. 6). C 1*s* spectra revealed peaks corresponding to C–C/C–H (284.8 eV), C–O (286.2 eV) and C=O (290.5 eV) at both 25 and − 35 °C (Fig. 6a, b). Cycling at 65 °C, however, introduced an additional peak at ~ 292.0 eV, assigned to O =C–O species, potentially yielded by decarboxylation of the DMC solvent under rising temperature (Fig. 6c) [54]. Analysis of F 1*s* spectra ascertained the components due to Na_x_PF_y_O_z_/Na_x_PF_y_ at ~ 688.0 eV and Na–F bonds at ~ 685.0 eV. The intensity of the P–F-signal was notably lower at − 35 °C compared to that at room temperature (Fig. 6d, e). This suppression can be explained by restricted ion diffusion at low temperature, stimulating SEI reorganization into a NaF-dominated structure that immensely reduces the desolvation energy barrier [67]. Conversely, P–F bond intensity at 65 °C recovered to levels comparable to ambient conditions (Fig. 6f), suggesting that phosphorus-containing fluorides were reformed by thermally induced electrolyte decomposition [68]. S 2*p* spectra at 25 °C displayed surface enrichment of oxidized sulfur species of Na_2_SO_3_, Na_2_SO_4_, which diminished after sputtering, alongside Na_2_S and Na_2_S_*x*_ (Fig. 6g). At − 35 °C, the migration of SO_3_ and Na_2_S hampered by restricted diffusion stabilized the sulfur speciation profile throughout etching (Fig. 6h) [69]. In contrast, in addition to bolstering electrode activity, elevated temperature encouraged the decomposition of SO_4_^2−^/SO_3_^2−^ into Na_2_S/Na_2_S_*x*_ and spurred the reduction of organic sulfur (Fig. 6i) [70].

**Fig. 7:**
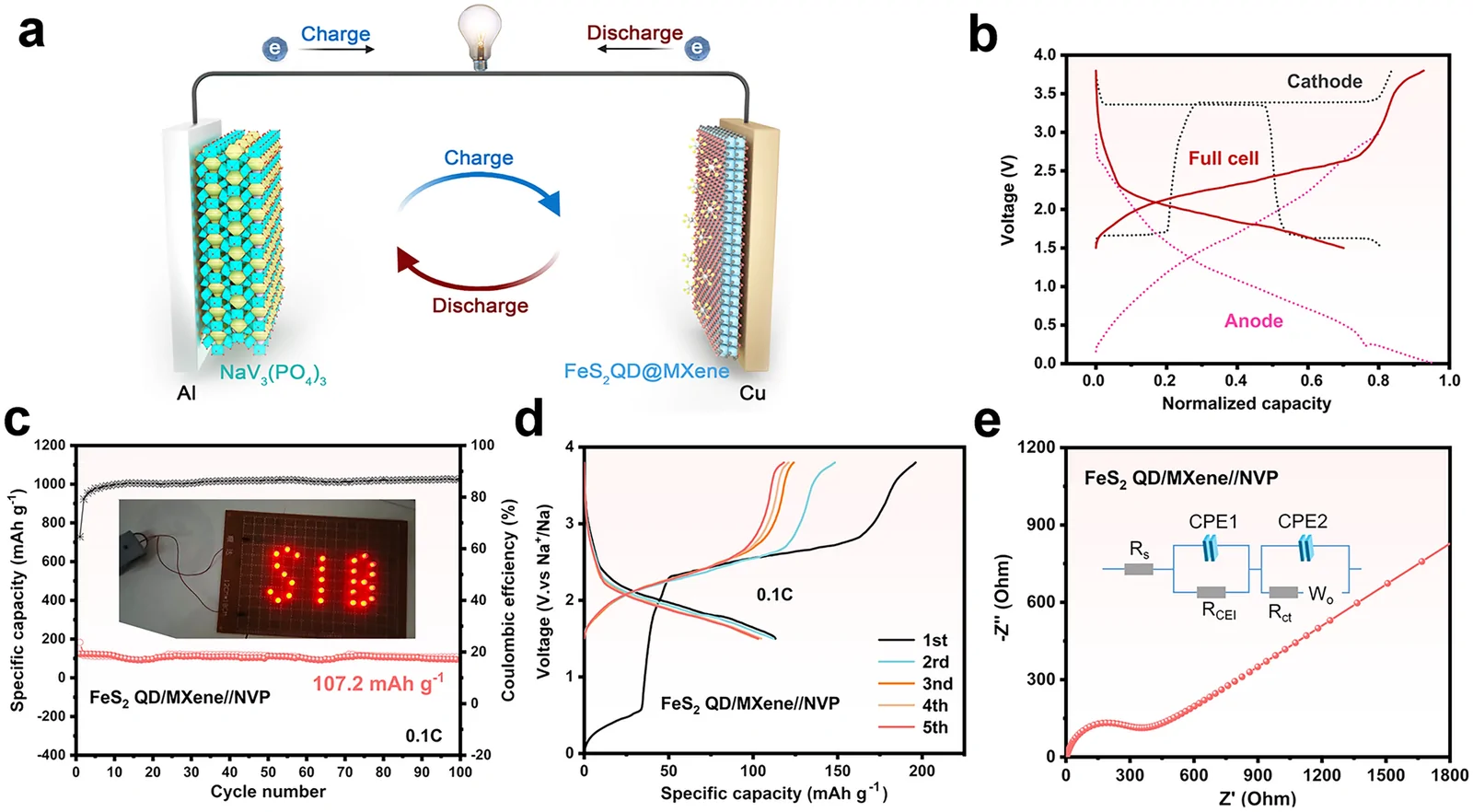
**a** Schematic illustration of the sodium full battery constructed by NVP cathode and FeS_2_ QD/MXene anode. **b** Charge and discharge curves, **c** cycling stability, **d** GCD profiles for the first five cycles at 0.1 C and **e** Nyquist pots of the FeS_2_ QD/MXene//NVP SIB

The ion-induced method presented in this study offers a viable approach for the in situ growth of various TMD-based quantum dots on 3D MXene substrates, such as CoS_2_ QD (Fig. S29). Leveraging the unique quantum structure, CoS_2_ QD/MXene and NiS_2_ QD/MXene deliver reversible capacities of 285.2 and 255.8 mAh g^−1^, respectively, after 100 cycles at a current of 0.1 A g^−1^, when used as SIB anodes. Additionally, DFT calculations indicate a significant increase in the Na⁺ adsorption energy, from − 0.53 eV in pristine SnS to − 1.51 eV in SnS QD (Fig. S30). This enhancement clearly demonstrates the crucial roles of quantum confinement and MXene hybridization in improving interfacial interactions across different metal sulfides. This work introduces an effective and general defect regulation strategy that holds promise for the development of superior TMD-based electrodes for sodium-ion batteries. To further demonstrate the practical application potential of the FeS_2_ QD/MXene electrode, a full cell was constructed using a FeS_2_ QD/MXene anode and a Na_3_V_2_(PO4)_3_ (NVP) cathode (Fig. 7a). The sodium-ion half-cell cycling performance, using an NVP cathode, is documented in Fig. S31, displaying a stable reversible capacity of 65 mAh g^−1^ after 100 cycles at 0.1 A g^−1^. To ensure a balanced capacity between the cathode and anode, the mass ratio of cathode-to-anode active materials in the full cell was maintained at approximately 3:1, with an anode mass loading of 1.1 mg. As shown in Fig. 7b, the output voltage range of the FeS_2_ QD/MXene//NVP SIB could be determined by the charge/discharge profiles of normalized capacity, and the range of working voltages were finally set as 1.5 ~ 3.8 V to ensure stable operation of the full cell. The FeS_2_ QD/MXene//NVP full cell exhibited relatively great stability (Fig. 7c), retaining 107.2 mAh g^−1^ of the capacity at 0.1 C after 100 cycles. Figure 7d presents the GCD curves for the first five cycles, indicating a well-preserved initial shape and suggesting an excellent match between the cathode and anode. Notably, Fig. S32 shows that at a temperature of − 35 °C, the full cell achieves a high energy density of 162.4 Wh kg^−1^ and a power density of 324.8 W kg^−1^. Subsequent EIS tests confirmed that the assembled full cell exhibits low interface impedance and superior ion transport performance (Fig. 7e), underscoring its potential for practical applications.

## Conclusions

In summary, an ion-diffusion-enhanced FeS_2_ QD/MXene anode with a unique delocalized electronic regions has been constructed for high sodium storage under wide temperatures. DFT calculations revealed that quantum-size effects enriched edge uncoordinated centers in FeS_2_, promoting charge delocalization at the atomic scale and optimizing electronic structure for accelerated charge transport. Moreover, the tightly connected of 3D skeleton and FeS_2_ QD produced excellent dispersibility and the highly stable electrode, which shortened the ions transport distance and facilitated the electrons transfer. Benefiting from the ideal edge electron concentration and strong interfacial coupling, the FeS_2_ QD/MXene anode delivers a high reversible capacity of 255.2 mAh g^−1^ at − 35 °C and 424.9 mAh g^−1^ at 65 °C after 100 cycles. Furthermore, the FeS_2_ QD/MXene//NVP full cell exhibits a 71.5% capacity retention (relative to that at 25 °C) after 100 cycles, indicating its high potential for practical use under wide-temperature conditions. This study not only provides insights into efficient Na^+^ storage and transport mechanisms but also offers a universal strategy for other TMD-based materials (e.g., CoS_2_ QD, NiS_2_ QD, and SnS QD). By shedding light on the design of quantum-scale structure and electron delocalization, this work makes it possible for developing high energy density sodium energy storage devices at wide temperature.

## Supplementary Information

Below is the link to the electronic supplementary material.

Supplementary file1 (DOCX 12017 KB)
