# Supplementary material for: Quantum-Size FeS2 with Delocalized Electronic Regions Enable High-Performance Sodium-Ion Batteries Across Wide Temperatures
Source: Nanomicro Lett. 2025 Jul 29;18:15. doi: 10.1007/s40820-025-01858-2 (PMC12307863; doi:10.1007/s40820-025-01858-2)
Supplement: Supplementary file 1 — Supplementary file1 (DOCX 12017 KB) [file 40820_2025_1858_MOESM1_ESM.docx]

Supporting Information for

**Quantum-Size FeS_2_ with Delocalized Electronic Regions Enable High-Performance Sodium-Ion Batteries Across Wide Temperatures**

Tianlin Li^1^, Danyang Zhao^1,2^*, Meiyu Shi^1^, Chao Tian^1^, Jie Yi^1^, Qing Yin^1,2^, Yongzhi Li^1,2^, Bin Xiao^1,2^, Jiqiu Qi^1,2^, Peng Cao^3^, Yanwei Sui ^1,2^*

^1^ China University of Mining and Technology, Xuzhou 221116, P. R. China

^2^ Jiangsu Province Engineering Laboratory of High-Efficient Energy Storage Technology and Equipment, School of Materials Science and Physics, China University of Mining and Technology, Xuzhou 221116, P. R. China

^3^ Department of Chemical & Materials Engineering, University of Auckland, Auckland 1142, New Zealand

*Corresponding authors. E-mail: [zhao_dy@cumt.edu.cn](mailto:zhao_dy@cumt.edu.cn) (Danyang Zhao); [wyds123456@outlook.com](mailto:wyds123456@outlook.com) (Yanwei Sui)

**S1 Experimental**

**S1.1 Materials characterization**

The morphology and microstructures are examined by TEM (JEOL JEM 2100F) operated and SEM equipped with an energy-dispersive X-ray spectroscopy (EDS) detector operated at 3KV. The TG analysis is performed using a TA analyzer. The crystal structure and phase composition are analyzed by X-ray diffraction (XRD, Bruker D8) with Cu Kα radiation (λ=0.1518 nm), between 10° and 80°. Raman scattering spectra is recorded with a Renishaw System 2000 spectrometer, and the wavelength of the laser is 532 nm. The crystal structure and phase composition are analyzed by X-ray diffraction (XRD, Bruker D8) with Cu Kα radiation (λ=0.1518 nm), between 10° and 80°. X-ray photoelectron spectroscopy (XPS, Thermo ESCALAB 250XI) is used to determine the chemical state of samples. The Micromeritics ASAP2460 analyzer is used to record nitrogen adsorption and desorption isotherms. The defect status of the samples is recorded by Bruker ELEXSYS-II E500. The X-ray absorption spectra (XAS) including X-ray absorption near-edge structure (XANES) and extended X-ray absorption fine structure (EXAFS) of the sample at Fe K-edge was collected at the Beamline of BL14B2 in SPring-8 (8 GeV, 100 mA), Japan.

**S1.2 Electrochemical measurement**

Anodes are prepared by mixing active materials, acetylene black, and PVDF with a mass ratio of 7:2:1 in NMP. The uniformly ground slurry is coated with designated thickness onto copper foil, drying at 80 ℃ for 12 h under vacuum. Assembling SIBs (CR2032) with Na foil as counter electrode to test the electrochemical properties of MXene, FeS_2_/MXene, Fe^2+^/MXene and FeS_2_ QD anodes. The electrolyte (under 25℃ and 65℃) is composed of 1M NaClO_4_ in ethylene carbonate (EC): dimethyl carbonate (DMC): ethyl methyl carbonate (EMC) = 1:1:1 wt % with 5.0 % fluoroethylene carbonate (FEC). And the electrolyte employed at -35℃ is commercially accessible, in which 1M NaClO_4_ in ethylene carbonate (EC): propylene carbonate (PC) = 1:1 wt %. PC and EC have high freezing points of -50℃, which can be used as excellent low-temperature electrolyte, as reported recently (Energy Storage Mater. 2024, 65, 103098; ACS Energy Lett. 2025, 10, 185-194). The galvanostatic charge/discharge (GCD) are carried out at 0.01-3.0 V, tested by Land cell measurement system (Wuhan Land Electronics Co., Ltd.). Cyclic voltammetry (CV) test is carried out at RST5080F electrochemical workstation. The GITT tests during charging/discharging with a current pulse of 200 mA g^−1^ for 10 min and a relaxation of 30 min within 0.01-3 V.

Cathodes are prepared by mixing NVP, acetylene black, and PVDF with a mass ratio of 7:2:1 in NMP. The uniformly ground slurry is coated with designated thickness onto aluminum foil, drying at 100 ℃ for 12 h under vacuum. Assembling SIBs (CR 2032) with Na foil as counter electrode to test the electrochemical properties of AC cathode. The electrolyte is composed of 1M NaPF_6_ in propylene carbonate (PC): ethyl methyl carbonate (EMC) = 1:1:1 vol % with 4.0 % fluoroethylene carbonate (FEC). The galvanostatic charge/discharge (GCD) are carried out at 1.5-3.8 V, tested by Land cell measurement system (Wuhan Land Electronics Co., Ltd.).

**S1.3 DFT calculation details**

The first-principles calculations are implemented in the vienna ab-initio simulation package (VASP) software [S1]. The electron states are represented by the project augmented wave (PAW) method with the Perdew-Burke-Ernzerhof (PBE) function under the generalized gradient approximation (GGA) [S2]. The cut-off energy is set to 350 eV, the force and energy convergence standards of each atom are respectively 0.05 eV Å^-1^ and 10^-6^ eV. A model of the FeS_2_ and FeS_2_ QD structure are achieved by a 15 Å vacuum layer along the z-axis.

The models of FeS_2_ and FeS_2_ QD are constructed. K-point network spaces of 3×3×1 and 4×4×1 are respectively used to optimize the structures. The construction is completed with the aid of the vaspkit module in the software [S3]. The Brillouin-zones were sampled with a k-point with Gamma point. A supercell of 3×3×1 is adopted, with a vacuum layer of 15 Å to remove the slab interaction in the z direction. The DFT+U energy functional to ensure the accuracy of the calculation [S4]. In addition, the density functional theory approximations (DFT-D4) as a more sophisticated dispersion model to process van der Waals (VDW) forces [S5]. Bader atoms in molecules (AIM) charges are calculated using the Henkelman algorithm [S6]. The climbing image nudged elastic band (CI-NEB) method is used to describe the diffusion energy barriers [S7].

To evaluate the Na adsorption capacity of different materials, **Eq. S1** is used to calculate the adsorption energy (E_ad_) [S8]:

$E_{\mathrm{ad}}=E_{structure+Na}-E_{\mathrm{structure}}$ (S1)

where $E_{Structure+Na}$ and $E_{\mathrm{Structure}}$ are respectively =the total energy of the Na atom adsorbed on the adsorptive substrate and the energy of corresponding substrate.

The change in the diffusion coefficient of metal ions with temperature can be calculated using the Arrhenius **Eq. S2** [S9, S10]:

$D=D_{0}\exp\left( -\frac{\Delta E_{b}}{\mathrm{KT}} \right)$ (S2)

where $D_{0}$,$\Delta E_{b}$, $K$ and $T$ are the pre-exponential factor, activation energy (diffusion barrier), Boltzmann’s constant and the absolute temperature.

The charge density differences were defined using the following equation:

$\Delta\rho\left( r \right)=\rho_{Na-structure}\left( r \right)-\rho_{\mathrm{Na}}\left( r \right)-\rho_{\mathrm{structure}}\left( r \right)$ (S3)

In which $\rho_{Na-structure}\left( r \right)-\rho_{\mathrm{Na}}\left( r \right)-\rho_{\mathrm{structure}}\left( r \right)$are the electronic charge distribution of the Na-absorbed substrate structure (FeS₂ and FeS₂ QD), the isolated Na and Na-free structure.

**S2 Supplementary Figures and Tables**

**
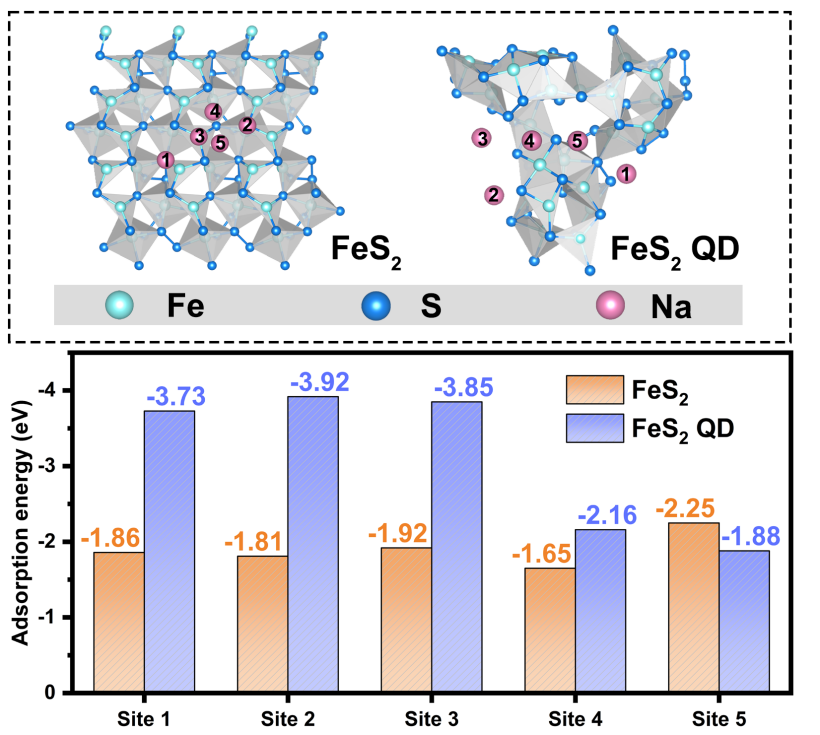
**

**Fig. S1** Na atoms adsorption energy on different sites of FeS_2_ and FeS_2_ QD


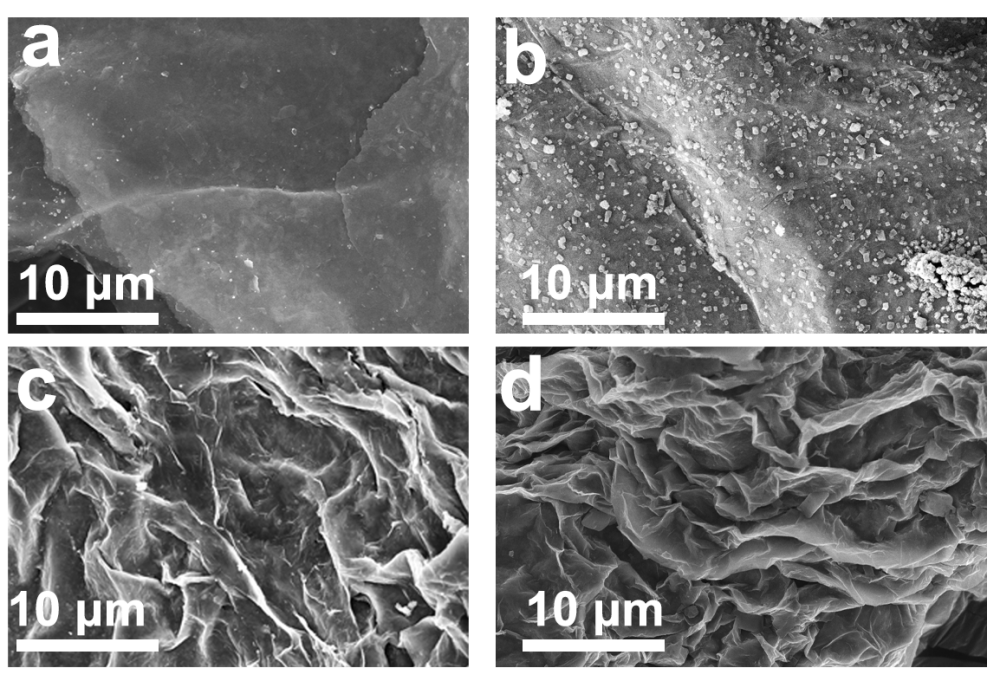


**Fig. S2** SEM images of **a** MXene, **b** FeS_2_/MXene, **c** Fe^2+^/MXene and **d** FeS_2_ QD/MXene


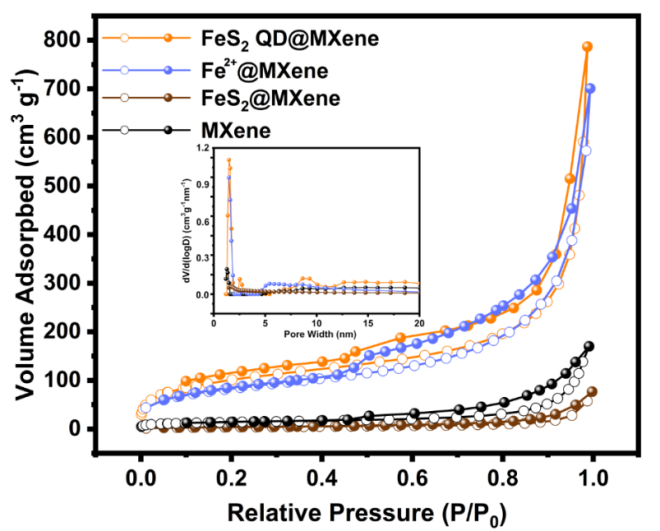


**Fig. S3** Nitrogen adsorption-desorption isotherms curves and pore size distribution of MXene, FeS_2_/MXene, Fe^2+^/MXene and FeS_2_ QD/MXene


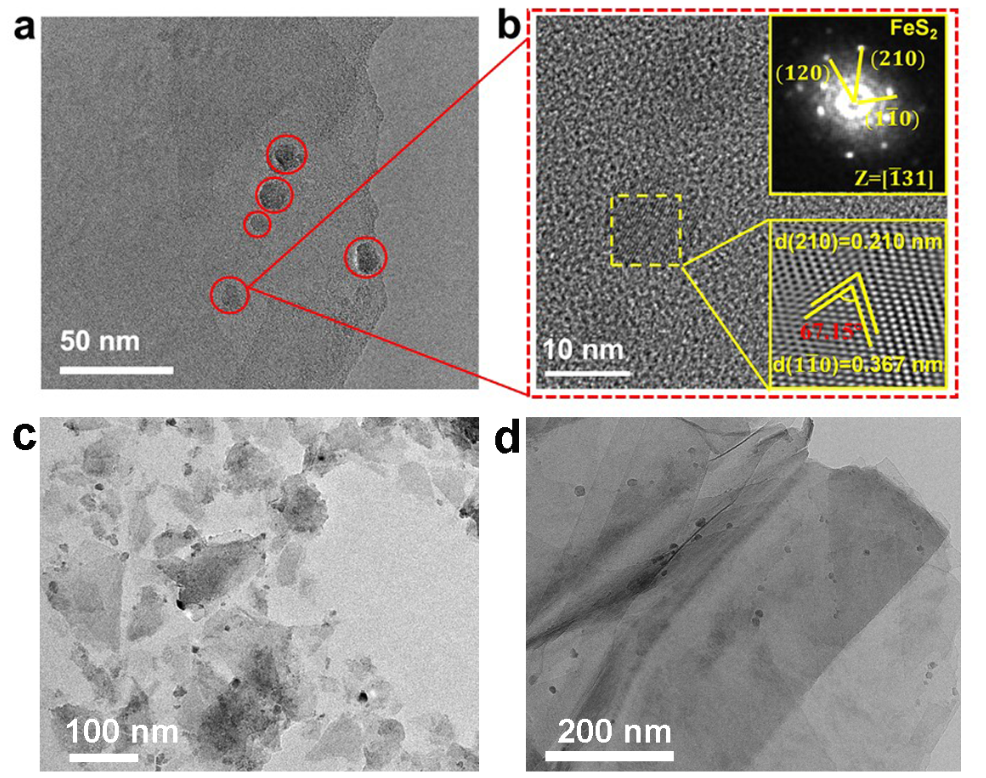
**Fig. S4** HRTEM images of FeS_2_ QD/MXene


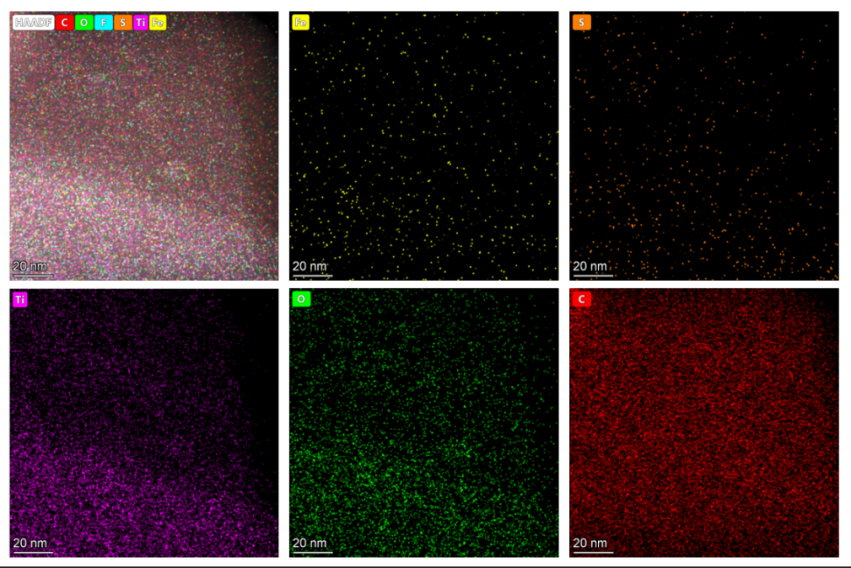


**Fig. S5** HAADF image and Fe, S, Ti, O and C elemental mapping of FeS_2_ QD/MXene


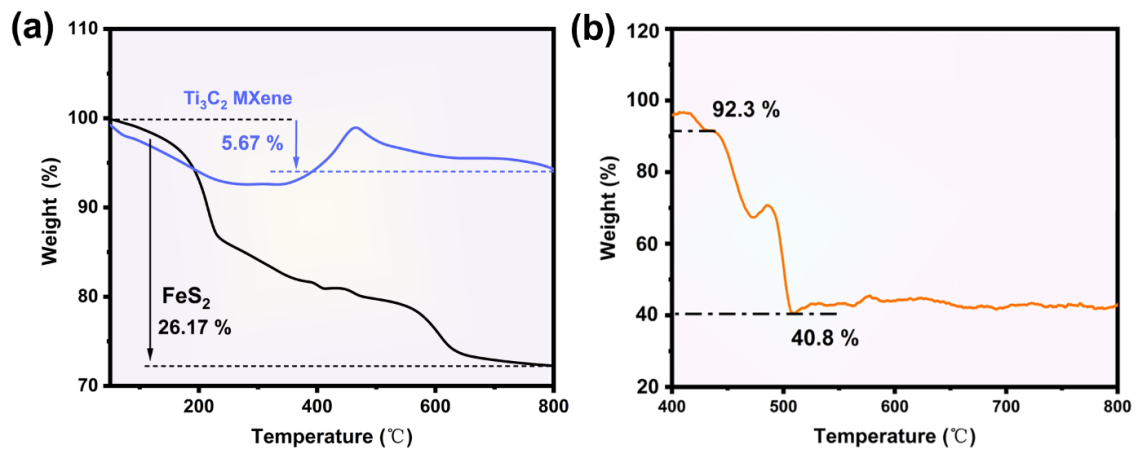


**Fig. S6** TG curve of **a** FeS_2_, Ti_3_C_2_ MXene and **b** FeS_2_ QD/MXene

Under the air conditioning, the weight loss of Ti_3_C_2_ MXene ($\mathrm{WT}i_{3}C_{2}$) achieved 5.67%. The weight loss observed at 100-300 ℃ was mainly attributed to desorption of the surface functional groups, then the weight increased at 300-450℃ is ascribed to the TiO_2_, and the final weight loss at 450-800℃ is due to releasing CO_2_ gas generated from the combustion of Ti_3_C_2_ MXene.^[11]^ Moreover, the weight loss of FeS_2_ ($\mathrm{WFe}S_{2}$) was determined to be 26.17%, and the was fully converted into Fe_3_O_4_ at 800℃.^[12]^ In addition, the weight loss of FeS_2_ QD/MXene composite was 51.5%, the calculated weight percentage of FeS_2_ in FeS_2_ QD/MXene was 36.6 %.


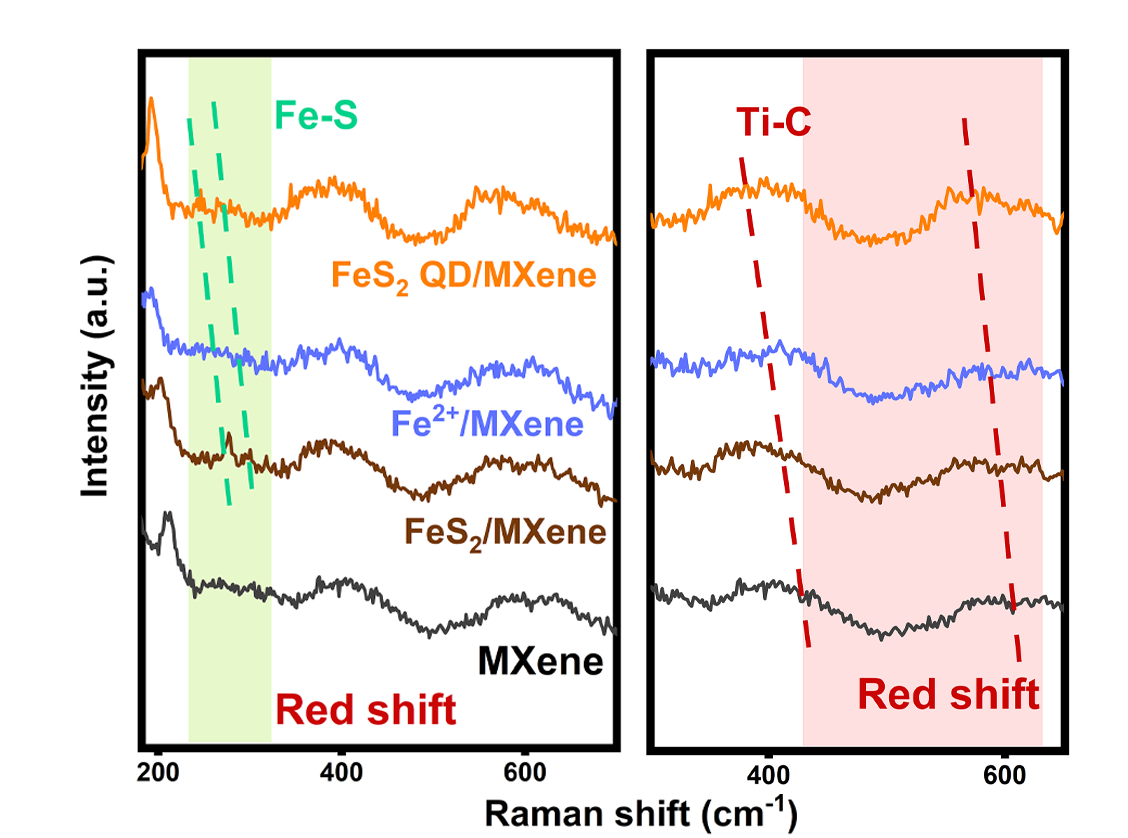


**Fig. S7** Raman spectra of MXene, FeS_2_/MXene, Fe^2+^/MXene and FeS_2_ QD/MXene


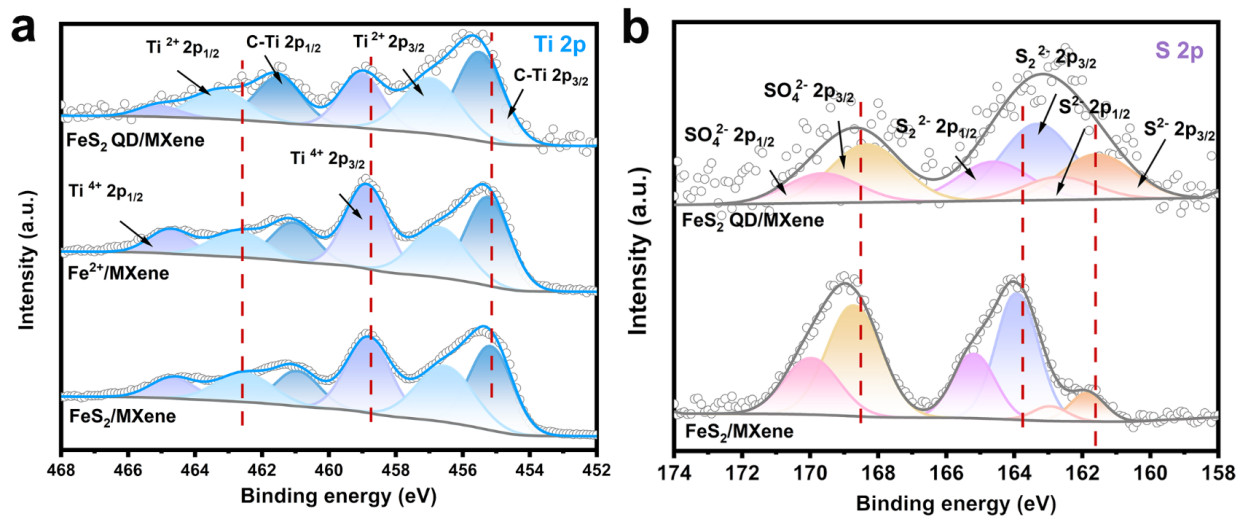
**Fig. S8** High-resolution XPS (**a**) Ti 2p (**b**) S 2p spectra


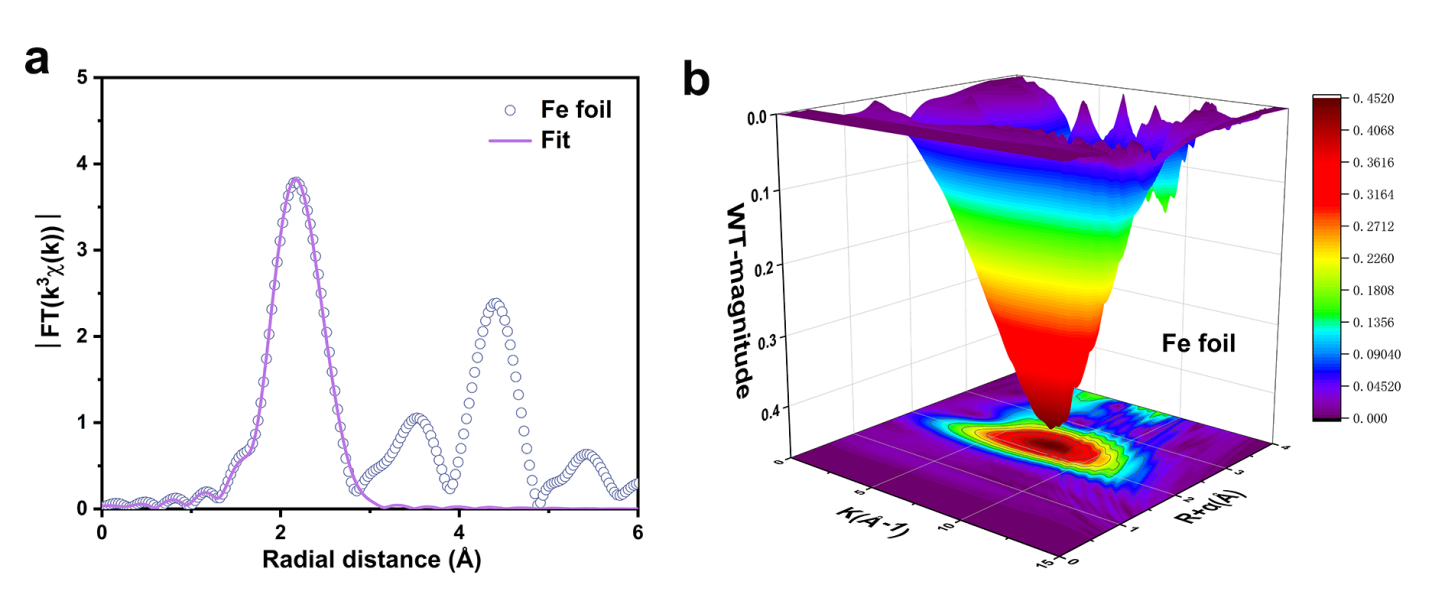


**Fig. S9** Wavelet transforms for the k^3^-weight EXAFS signals of FeS_2_

**
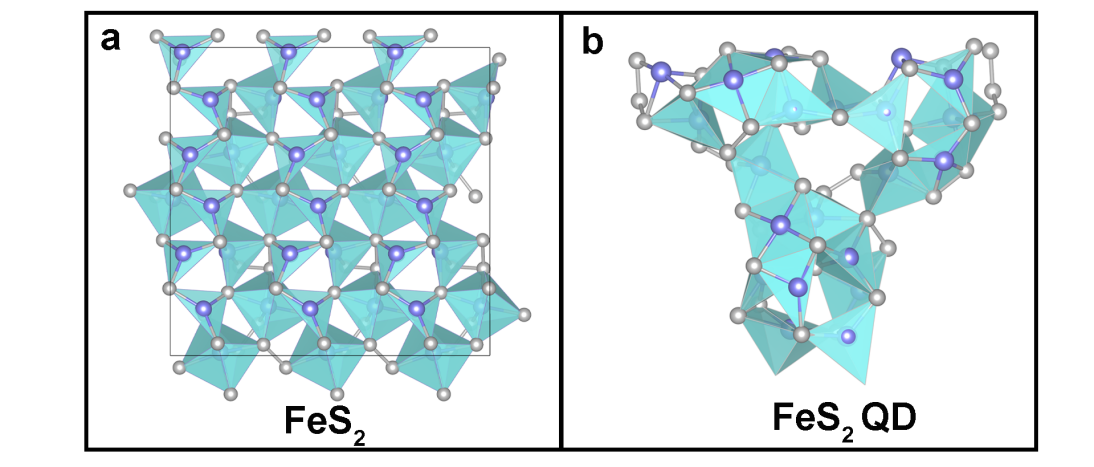
**

**Fig. S10** Structure of FeS_2_ and FeS_2_ QD structures


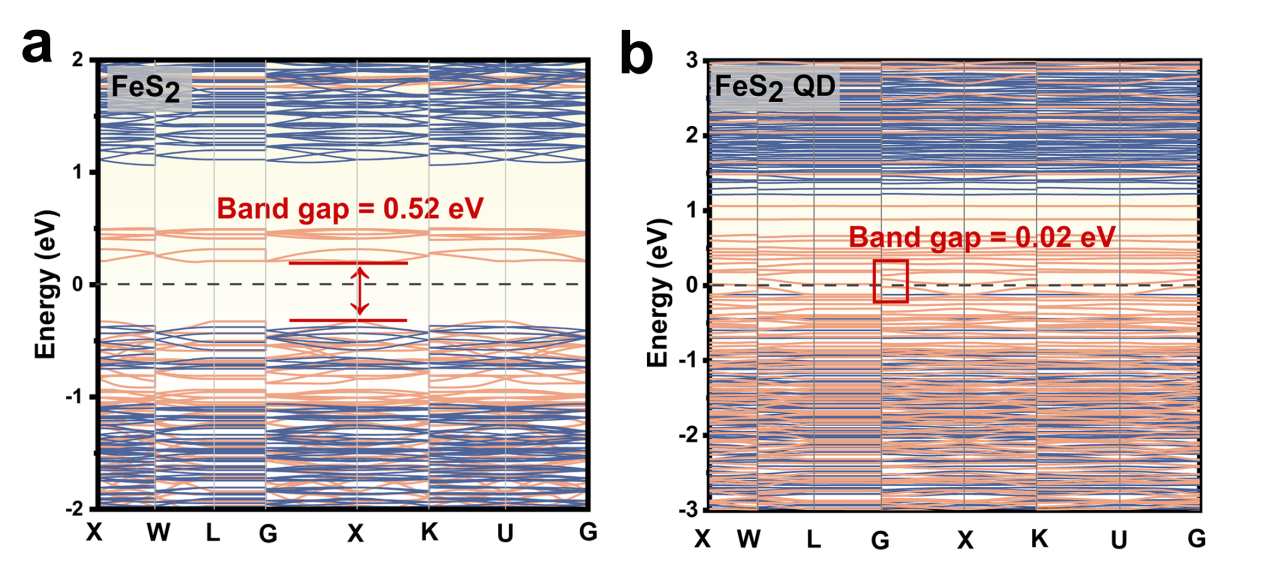


**Fig. S11** Band structure diagrams of FeS_2_ and FeS_2_ QD structures

**
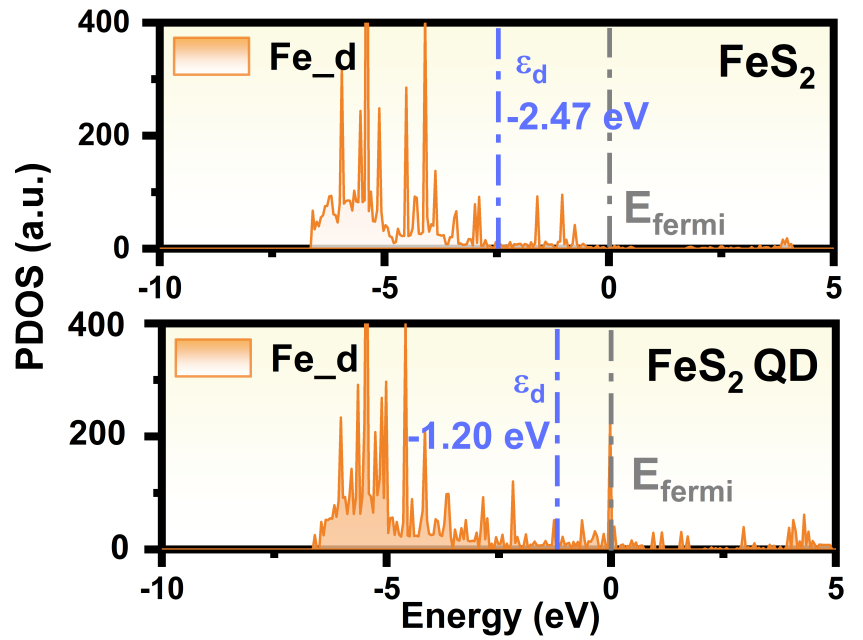
**

**Fig. S12** D-band center for Fe atoms of FeS_2_ and FeS_2_ QD structures


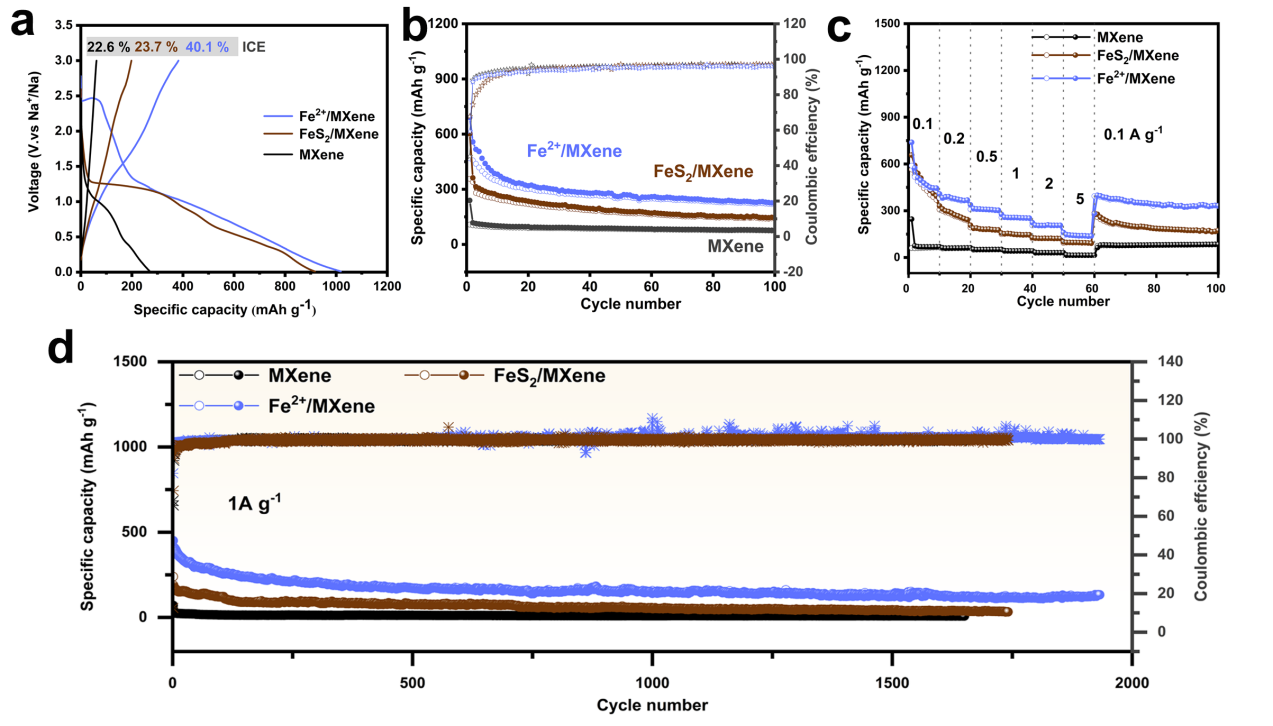
**Fig. S13 a** GCD profiles for first five cycles at 0.1 A g^-1^, **b** cycle performance at 0.1 A g^-1^, **c** rate capability at increasing current densities of MXene, FeS_2_/MXene and Fe^2+^/MXene anodes


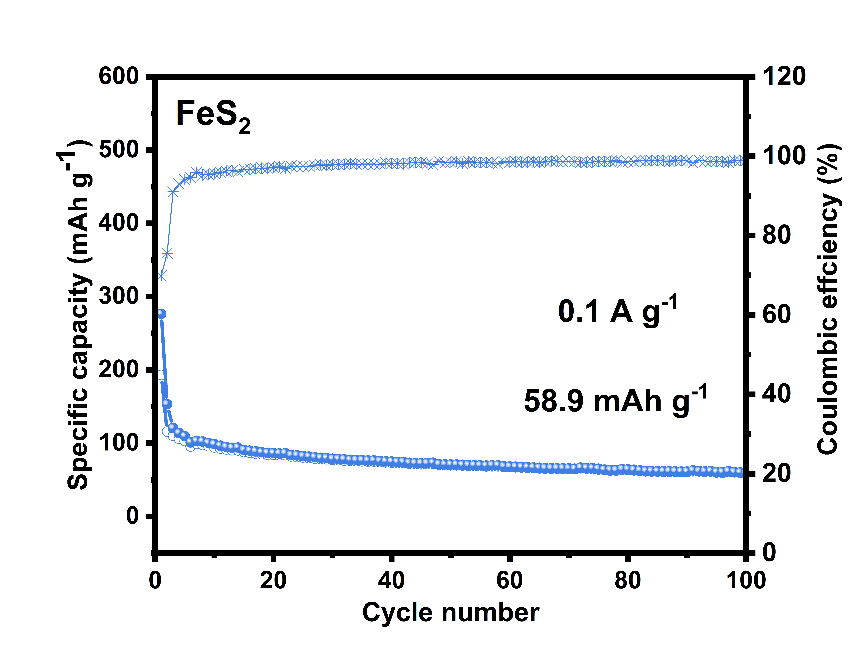


**Fig. S14** Cycle performance at 0.1 A g^-1^ of FeS_2_ electrode


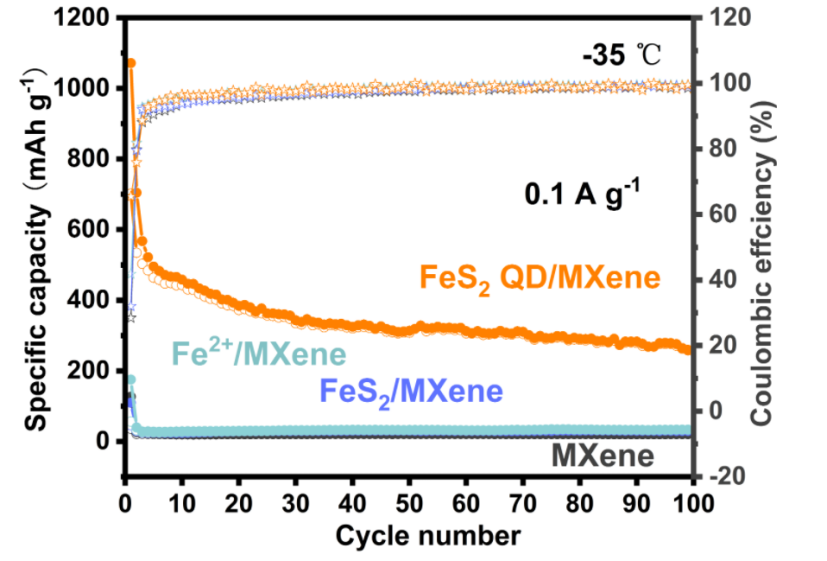


**Fig. S15** Cycling performance of MXene, FeS_2_ /MXene and Fe^2+^/MXene anodes at -35 ℃


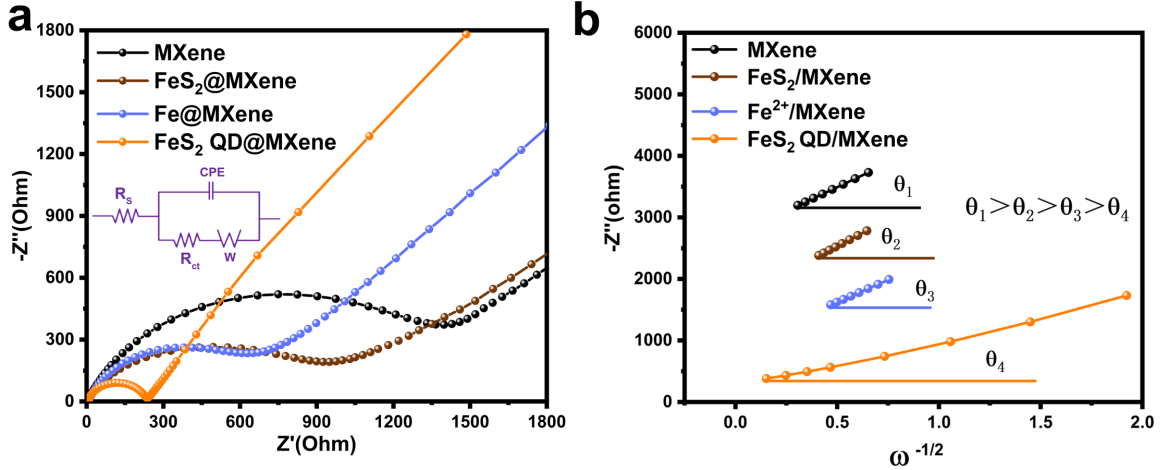
**Fig. S16 a** Nyquist pots and **b** relationship between Zre and ω^-1/2^ of MXene, FeS_2_/MXene, Fe^2+^/MXene and FeS_2_ QD/MXene anodes


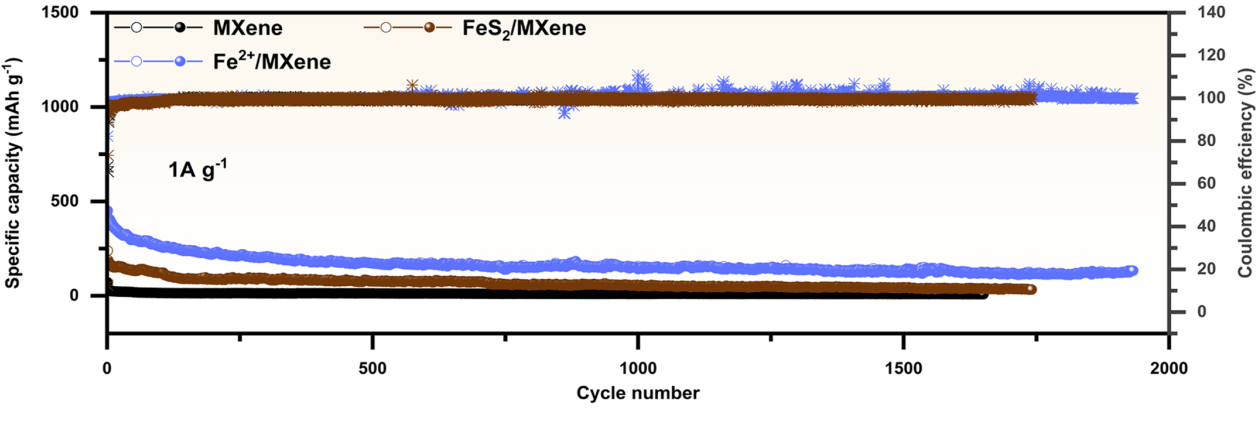


**Fig. S17** Long-term cycling performance of MXene, FeS_2_/MXene and Fe^2+^/MXene anodes


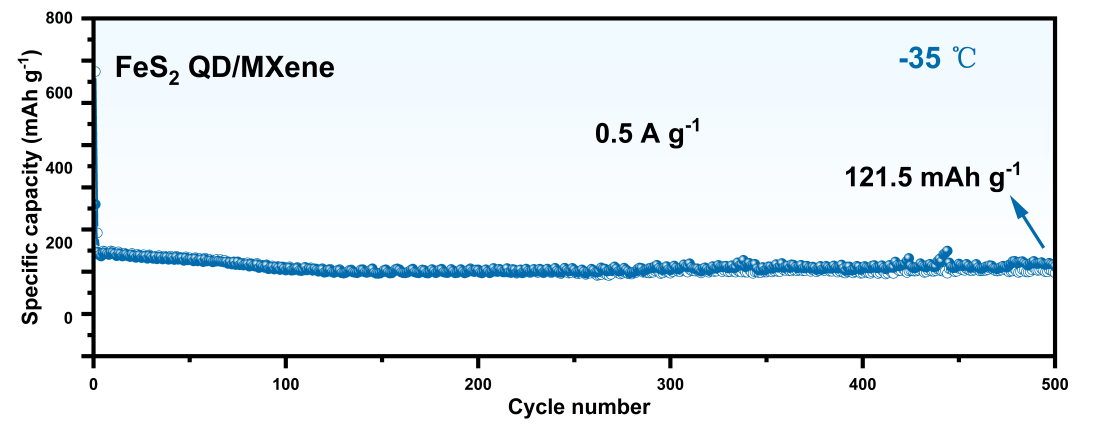


**Fig. S18** Long-term cycling performance of FeS_2_ QD/MXene anode at -35 ℃


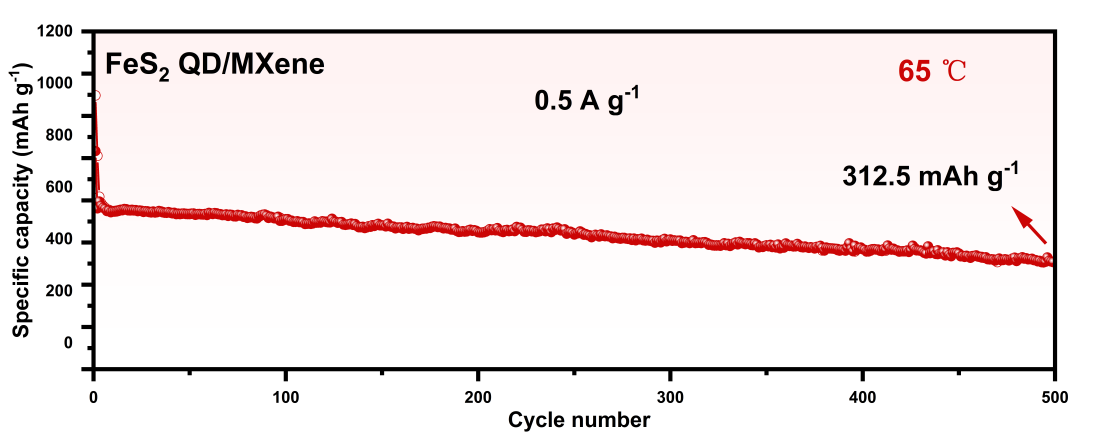


**Fig. S19** Long-term cycling performance of FeS_2_ QD/MXene anode at 65 ℃


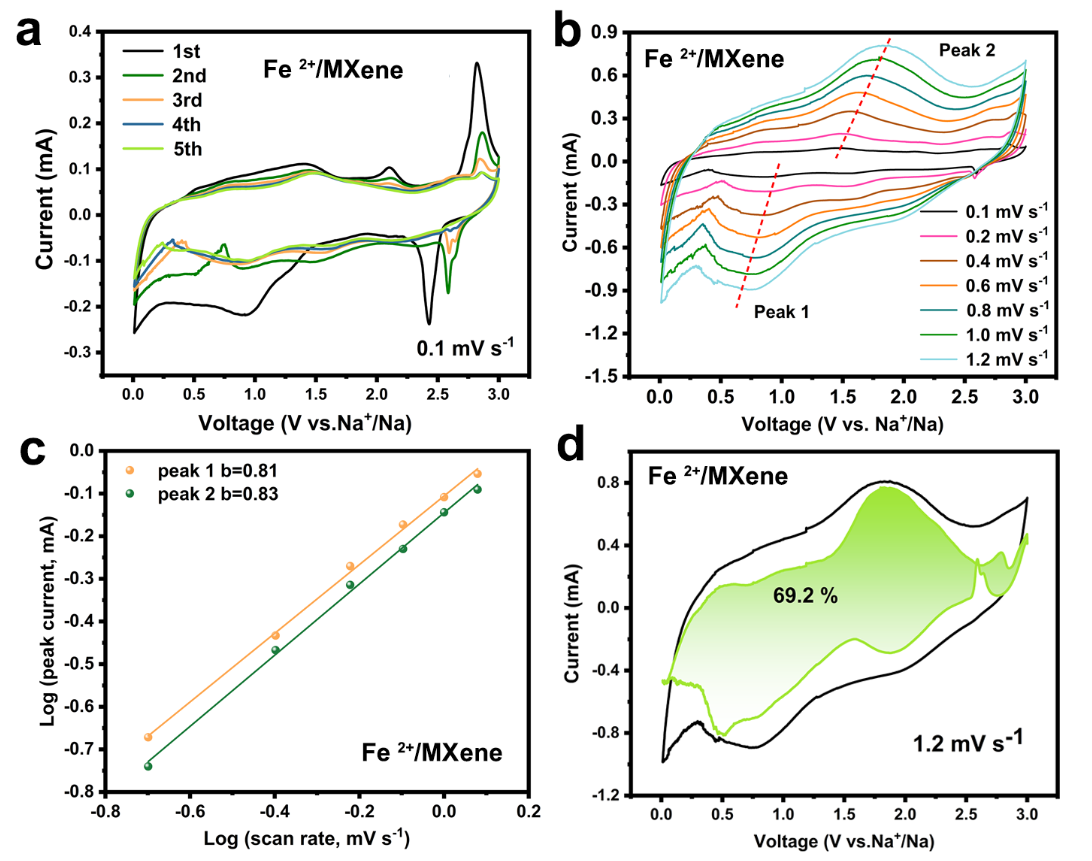


**Fig. S20 a** CV curves at 0.1 mV s^-1^ in 0.01-3.0 V, **b** CV curves at 0.1 to 1.2 mV s^-1^, **c** b-value of redox peaks and **d** pseudocapacitance contribution at 1.2 mV s^-1^ of Fe^2+^/MXene electrode


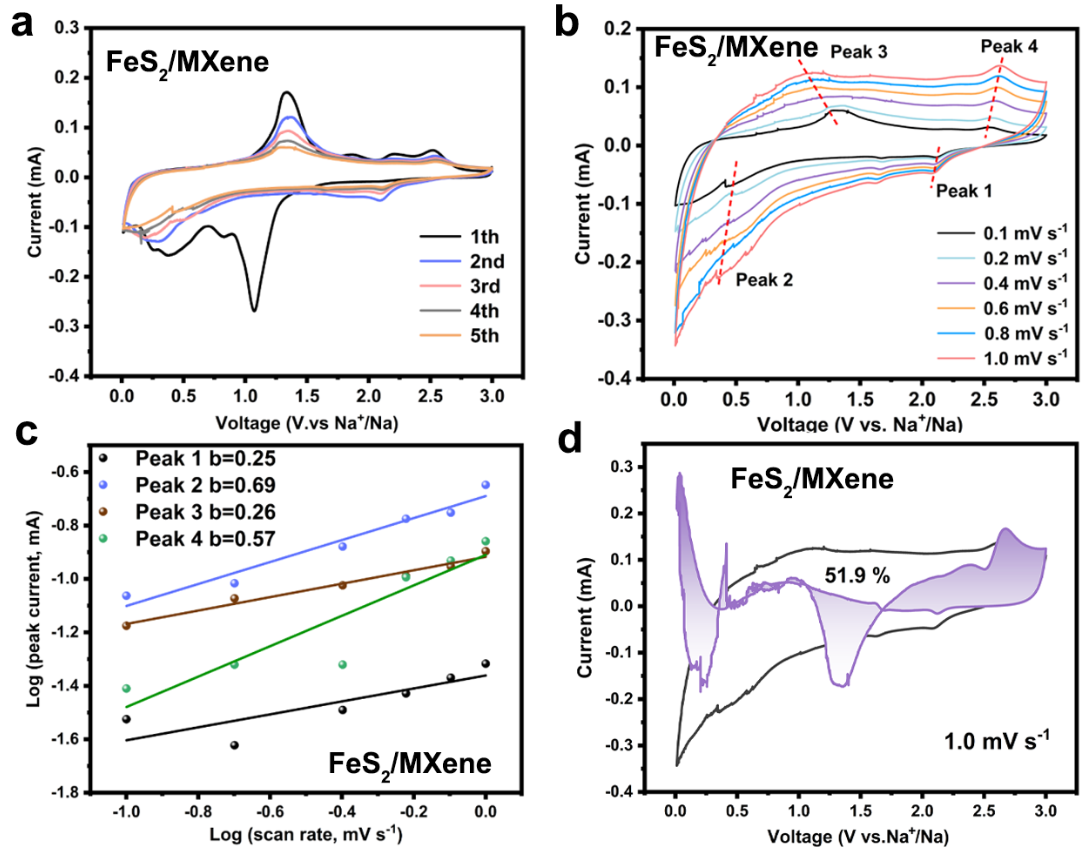


**Fig. S21 a** CV curves at 0.1 mV s^-1^ in 0.01-3.0 V, **b** CV curves at 0.1 to 1.0 mV s^-1^, **c** b-value of redox peaks and **d** pseudocapacitance contribution at 1.0 mV s^-1^ of FeS_2_/MXene electrode

**
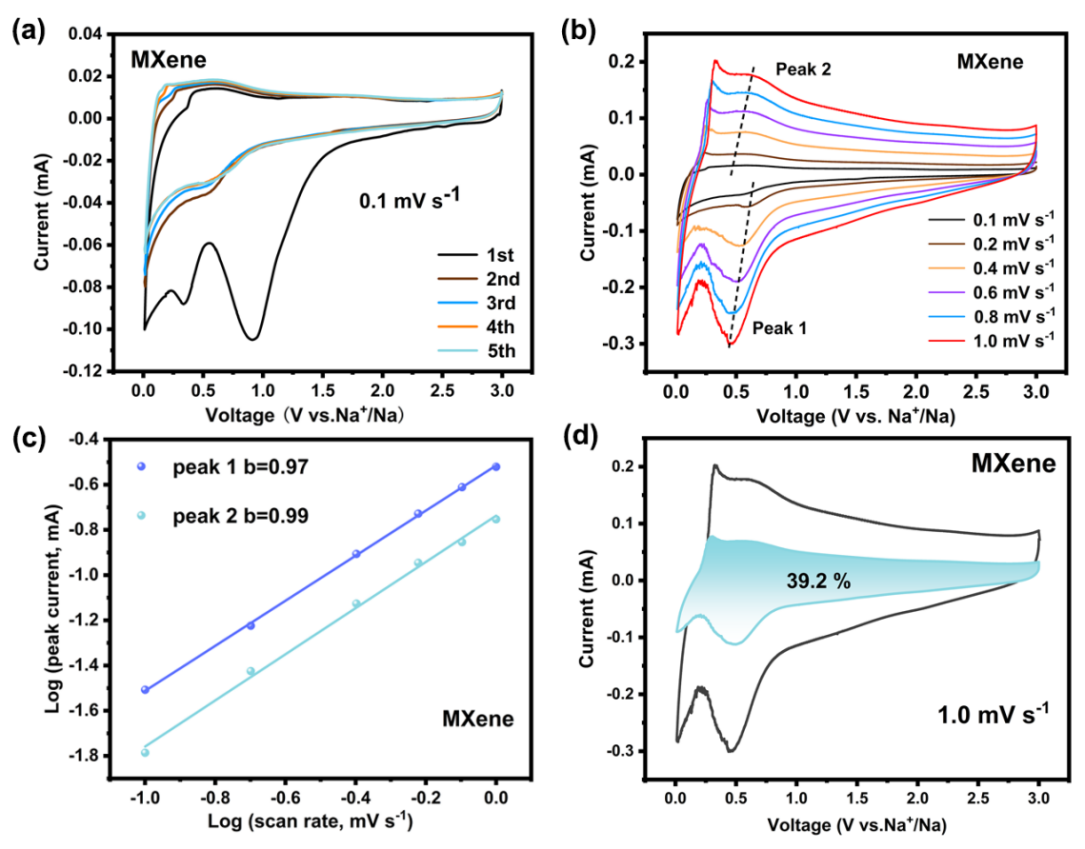
**

**Fig. S22 a** CV curves at0.1 mV s^-1^ in 0.01-3.0 V, **b** CV curves at 0.1 to 1.0 mV s^-1^, **c** b-value of redox peaks and **d** pseudocapacitance contribution at 1.0 mV s^-1^ of MXene electrode


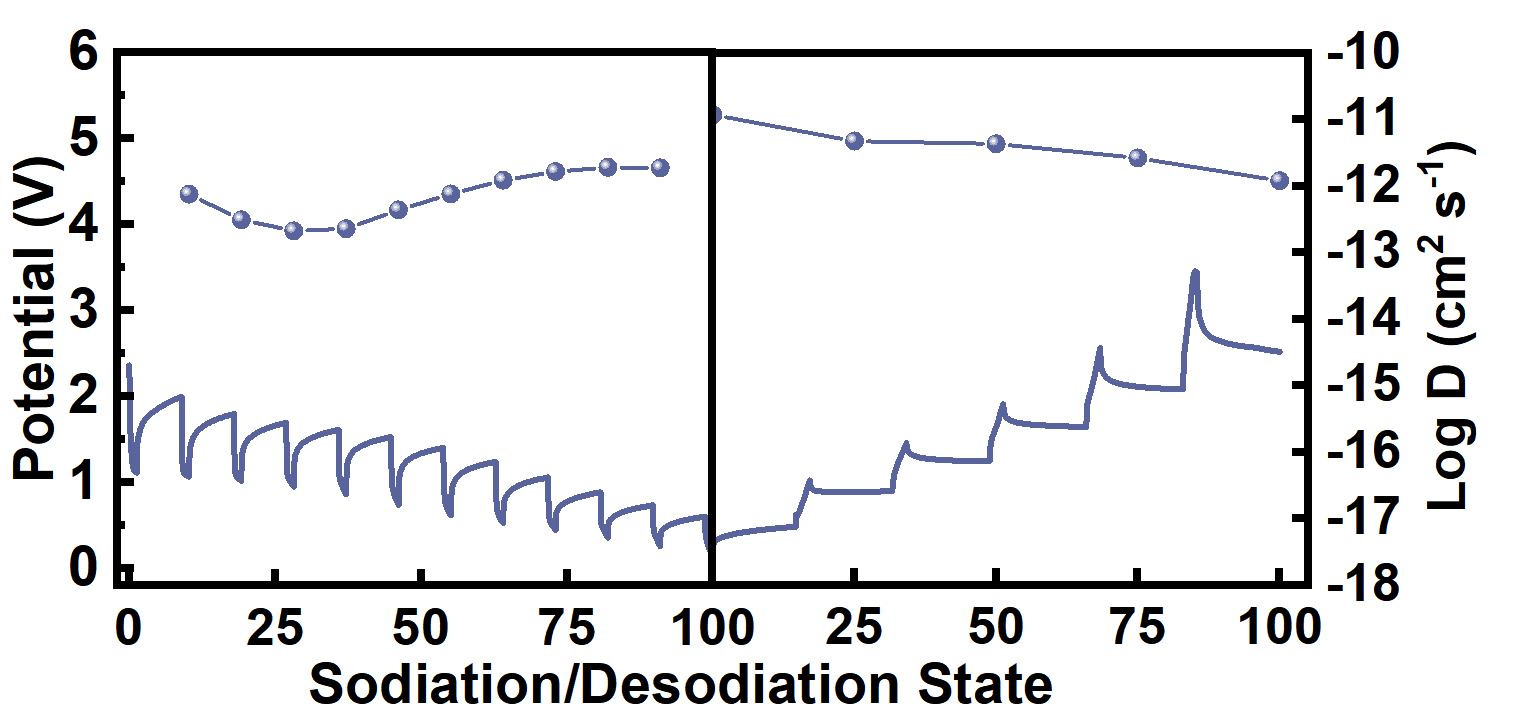


**Fig. S23** GITT potential profiles and Na^+^ ions diffusion system of FeS_2_ electrode


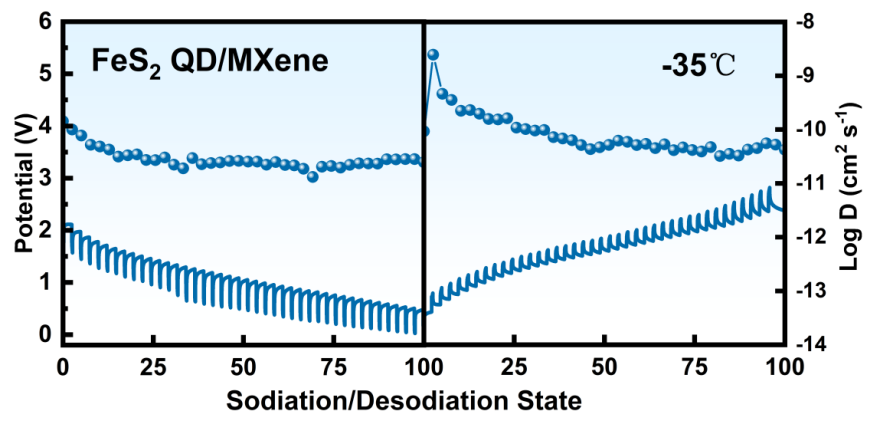


**Fig. S24** GITT potential profiles and Na^+^ ions diffusion system of FeS_2_ QD/MXene electrode at -35 ℃


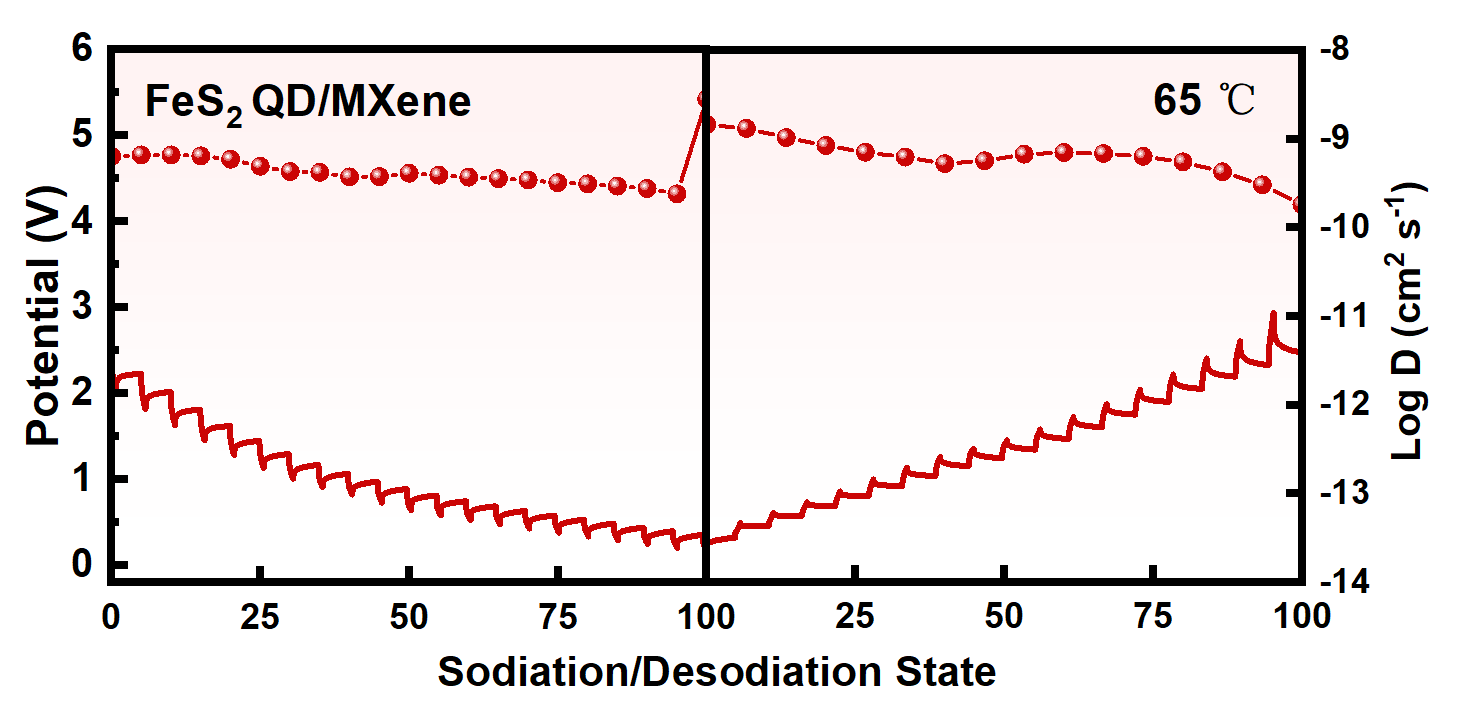


**Fig. S25** GITT potential profiles and Na^+^ ions diffusion system of FeS_2_ QD/MXene electrode at 65 ℃


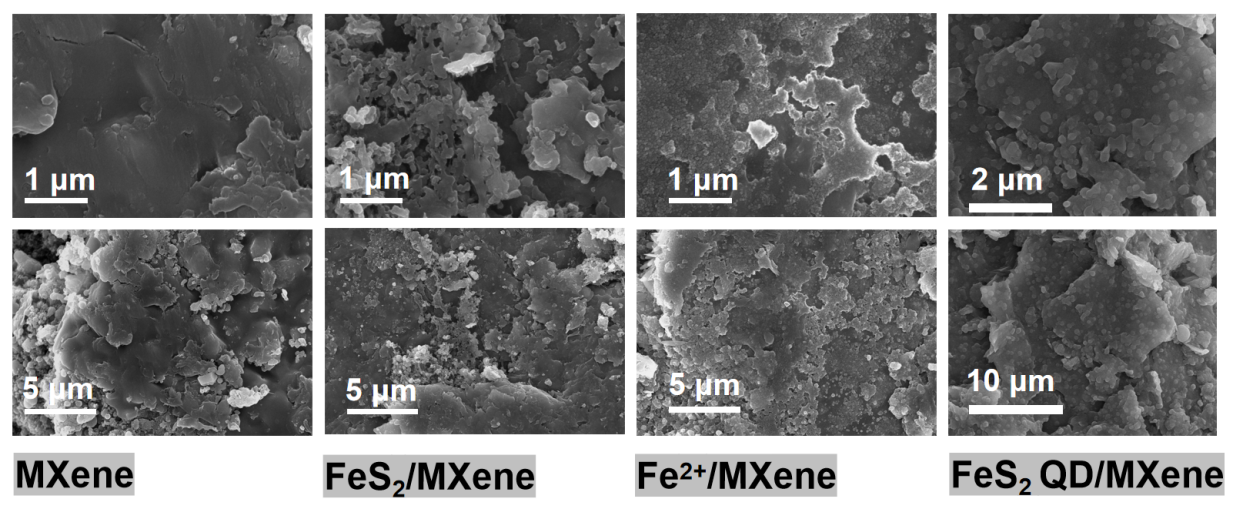


**Fig. S26** SEM images of MXene, FeS_2_/MXene, Fe^2+^/MXene and FeS_2_ QD/MXene electrodes after cycling for the 20 cycles at 0.1 A g^-1^


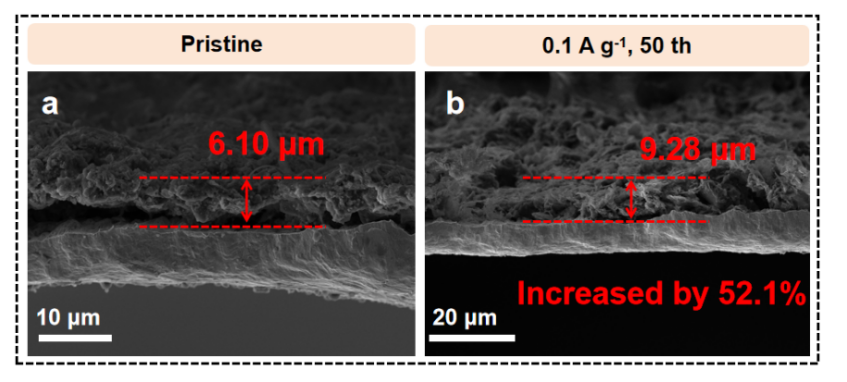


**Fig. S27** Cross-sectional SEM images of FeS_2_/MXene electrode **a** before cycling and **b** after cycling for the 50 cycles at 0.1 A g^-1^


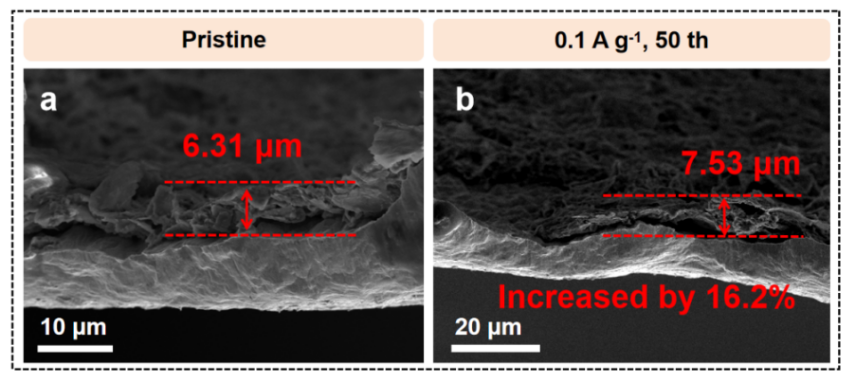


**Fig. S28** Cross-sectional SEM images of FeS_2_ QD@MXene electrode **a** before cycling and **b** after cycling for the 50 cycles at 0.1 A g^-1^

**
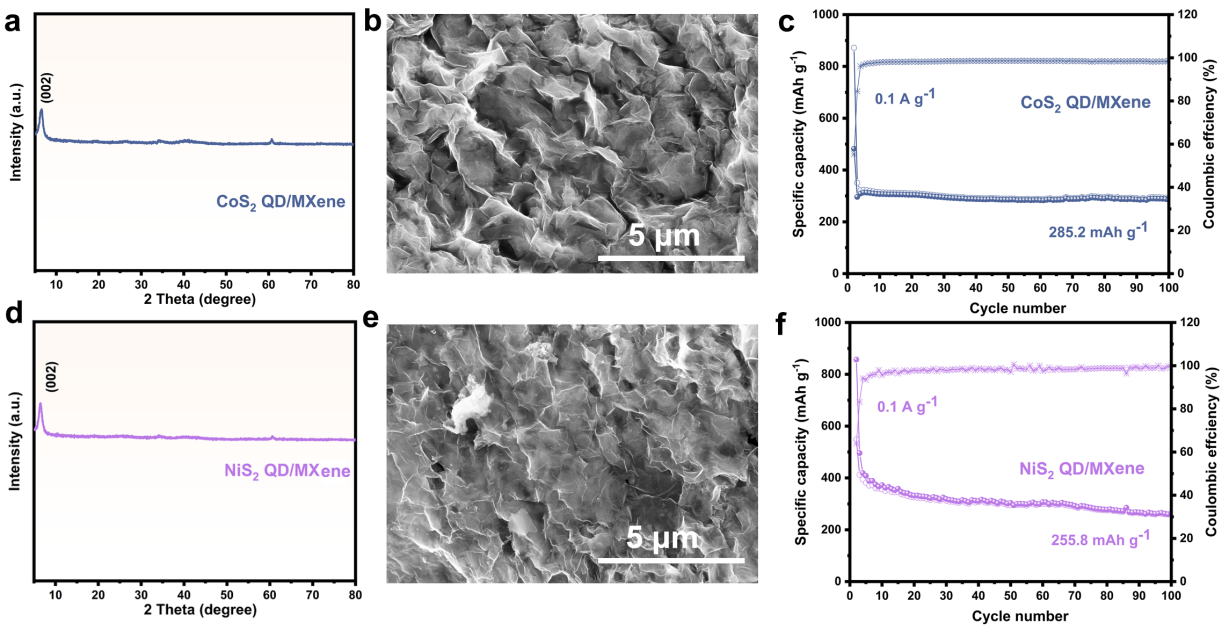
****Fig. S29** **a** XRD spectra, **b** SEM image and **c** cycle performance of CoS_2_ QD/MXene anode. **d** XRD spectra, **e** SEM image and **f** cycle performance of NiS_2_ QD/MXene anode


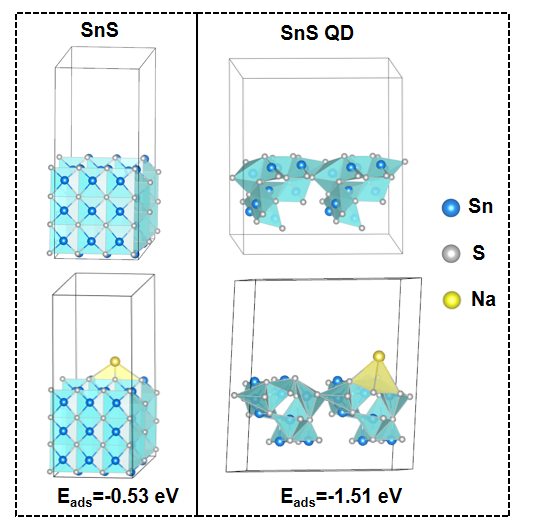


**Fig. S30** Na atom adsorption energy on SnS and SnS QD

**
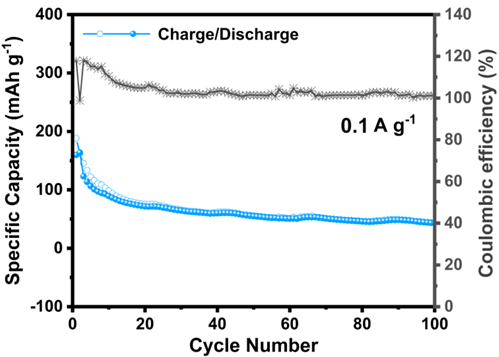
**

**Fig. S31** Cycle performance at 0.1A g^-1^ of NVP cathode


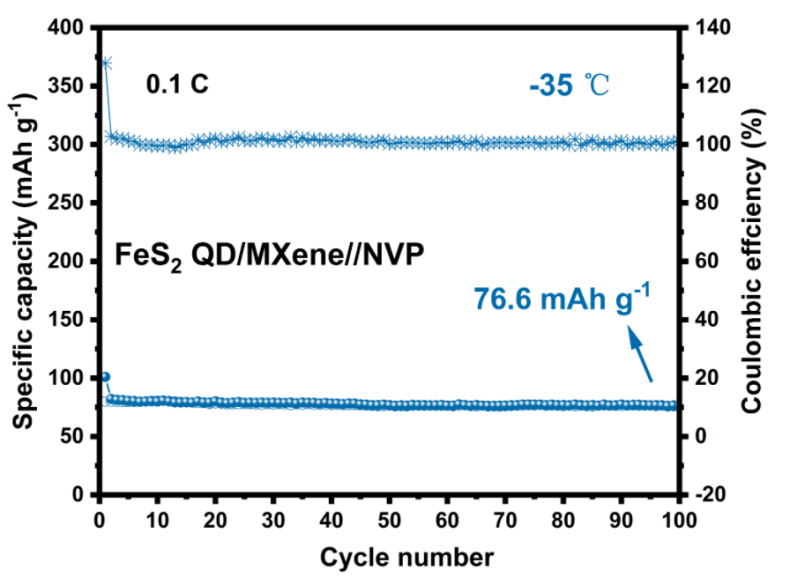


**Fig. S32** Cycling stability performance at 0.1 C of FeS_2_ QD/MXene//NVP SIB under -35 ℃

**Table S1** EXAFS fitting parameters at the Fe K-edge for various samples

|  | Shell | CN*^a^* | R*^b^*(Å) | σ^2^*^c^*(Å^2^) | ΔE_0_*^d^*(eV) | R factor |
| --- | --- | --- | --- | --- | --- | --- |
| Fe-foil | Fe-Fe_1_ | 8.00 | 2.45±0.01 | 0.0045 | 5.0 | 0.006 |
|  | Fe-Fe_2_ | 6.00 | 2.84±0.01 | 0.0041 |  |  |
| FeS_2_ QD/MXene | Fe-S | 5.25 | 2.32±0.08 | 0.0097 | -2.3 | 0.017 |
|  | Fe-S | 1.41 | 3.40±0.04 | 0.00107 |  |  |
|  | Fe-O-Ti | 2.09 | 3.79±0.02 | 0.0094 |  |  |
| FeS_2_/MXene | Fe-S | 6.56 | 2.22±0.03 | 0.0112 | -2.6 | 0.015 |
|  | Fe-S | 2.44 | 3.37±0.07 | 0.0086 |  |  |

^a^CN: coordination numbers; ^b^R: bond distance; ^c^σ^2^: Debye-Waller factors; ^d^ΔE_0_: the inner potential correction. R factor: goodness of fit. Error bounds that characterize the structural parameters obtained by EXAFS spectroscopy were estimated as CN±20%; R ± 1%; σ^2^ ± 20%

**Table S2** Electrode resistances obtained from the equivalent circuit fitting

| Electrode | V_OC_ (V) | R_e_ (Ω) | R_ct_ (Ω) | Warburg factor σ | D_Na_^+^ (cm^2^ S^-1^) |
| --- | --- | --- | --- | --- | --- |
| MXene | 2.58 | 3.51 | 866.7 | 1634.0 | 6.60×10^-9^ |
| FeS_2_/MXene | 2.32 | 5.49 | 734.1 | 1105.2 | 4.72×10^-9^ |
| Fe^2+^/MXene | 2.41 | 6.86 | 628.5 | 1396.0 | 2.95×10^-9^ |
| FeS_2_ QD/MXene | 2.52 | 7.79 | 193.0 | 702.3 | 1.17×10^-8^ |

The diffusion rate of lithium ion can be calculated by using the low-frequency data according to the following formula:

$D_{\mathrm{Na}^{+}}$ = $\frac{R^{2}T^{2}}{2A^{2}n^{2}F^{4}C_{Na}^{2}\sigma^{2}}$ (S4)

Where $D_{\mathrm{Na}^{+}}$ is the Na^+^ ions diffusion efficiency, $R$ is the ideal gas constant, $T$ is the absolute temperature (298.15 K), $A$ is the surface area of the electrode (π×0.7^2^), $n$ is the number of transferred electrons per FeS_2_ molecule during oxidization process (n=4), $F$ is the Faraday constant (96485 C), $C$ is the Na^+^ ions concentration (1.0×10^-6^ mol cm^-3^), as well as $\sigma$ is the Warburg factor which could be obtain from **Eq. S3**.

| Materials | Current density  (mA g^-1^) | Cycle numbers | Reversible capacity  (mAh g^-1^) | References |
| --- | --- | --- | --- | --- |
| CoS/C | 200 | 100 | 373.2 | [S13] |
| MoS_2-x_Se_x_/GF | 200 | 500 | 172 | [S14] |
| MoS_0.74_Se_1.26_/NC | 1000 | 800 | 278 | [S15] |
| FeS/NC | 1000 | 500 | 326 | [S16] |
| CoS/MXene | 2000 | 1700 | 267 | [S17] |
| FeS/CFs | 1000 | 400 | 283 | [S18] |
| CoSe/HPCNF | 1000 | 550 | 319 | [S19] |
| ZnSe/CoSe/NC | 1000 | 900 | 303.9 | [S20] |
| SnS/rGO | 500 | 200 | 370 | [S21] |
| **FeS_2_ QD/MXene** | **100/1000** | **100/2500** | **463.8/370.1** | **This work** |

**Table S3** Comparison on electrochemical performance at room temperature (25℃) between our work and resent TMDs-based sodium ion storage

**Table S4** Comparison on electrochemical performance at wide-temperature between our work and recent TMDs-based sodium ion storage (It is important to note that our work focuses on optimizing the electrode materials, where the electrolytes used are common ester based electrolytes. Therefore, we only compare recent work on electrodes optimization, and the latest research results under wide-temperature have not been compared.)

|  | Temperature (℃) | Current density  (mA g^-1^) | Cycle numbers | Reversible capacity  (mAh g^-1^) | References |
| --- | --- | --- | --- | --- | --- |
| NbSSe | 0 | 200 | 500 | 136 | [S22] |
| C_3_N_4_ | -20 | 100 | 500 | 118 | [S23] |
| Bi@C | -40 | 100 | 10 | 247 | [S24] |
| Ti_0.88_Nb_0.88_O_4-x_@C | -40 | 250 | 500 | 99 | [S25] |
| NFPP@C | -40 | 200 | 40 | 173 | [S26] |
| ZnSe@NCNFs | -20 | 1000 | 1000 | 123 | [S27] |
| KTOP | -35 | 450 | 550 | 90 | [S28] |
| MoS_2_@MXene | -30 | 50 | 100 | 180 | [S29] |
| NaTi_2_ (PO_4_)_3_/C | -20 | 200 | 100 | 125 | [S30] |
| P-Bi@C-700 | 60 | 50 | 50 | 370.3 | [S31] |
| Lignin | 60 | 200 | 60 | 262 | [S32] |
| NaTi_2_ (PO_4_)_3_ | 60 | 1000 | 20 | 136 | [S33] |
| **FeS_2_ QD/MXene** | **-35** | **100/500** | **100/500** | **255.2/121.5** | **This work** |
| **FeS_2_ QD/MXene** | **65** | **100/500** | **100/500** | **424.9/312.5** | **This work** |

**Supplementary Figures**

1. G. Kresse, J. Furthmüller, Efficiency of ab-initio total energy calculations for metals and semiconductors using a plane-wave basis set. Comput. Mater. Sci. **6**(1), 15–50 (1996). <https://doi.org/10.1016/0927-0256(96)00008-0>
2. G. Kresse, J. Hafner, Ab initio molecular-dynamics simulation of the liquid-metal-amorphous-semiconductor transition in germanium. Phys. Rev. B Condens. Matter **49**(20), 14251–14269 (1994). <https://doi.org/10.1103/physrevb.49.14251>
3. V. Wang, N. Xu, J.-C. Liu, G. Tang, W.-T. Geng, VASPKIT: a user-friendly interface facilitating high-throughput computing and analysis using VASP code. Comput. Phys. Commun. **267**, 108033 (2021). <https://doi.org/10.1016/j.cpc.2021.108033>
4. H. Hajiyani, R. Pentcheva, Surface termination and composition control of activity of the Co*_x_*Ni_1–_*_x_*Fe_2_O_4_(001) surface for water oxidation: insights from DFT+*U* calculations. ACS Catal. **8**(12), 11773–11782 (2018). <https://doi.org/10.1021/acscatal.8b00574>
5. E. Caldeweyher, S. Ehlert, A. Hansen, H. Neugebauer, S. Spicher et al., A generally applicable atomic-charge dependent London dispersion correction. J. Chem. Phys. **150**(15), 154122 (2019). <https://doi.org/10.1063/1.5090222>
6. L. Dong, X. H. Xian, T. F. Lin, D. X. Kai, C. J. Xiang et al., Graphical Abstract. Angew. Chem. Int. Ed. **61**, e202203698 (2022). <https://doi.org/10.1002/anie.202282611>
7. C. Chen, H. Ying, M.Z. Yue, M.W. Lu, X.Z. Peng et al., Experimental design and theoretical evaluation of nitrogen and phosphorus dual-doped hierarchical porous carbon for high-performance sodium-ion storage. J. Mater. Sci. Technol. **76**, 11-19 (2021). <https://doi.org/10.1016/j.jmst.2020.11.014>
8. J. Lin, C. Zeng, X. Lin, C. Xu, C.-Y. Su, CNT-assembled octahedron carbon-encapsulated Cu_3_P/Cu heterostructure by *in situ* MOF-derived engineering for superior lithium storage: investigations by experimental implementation and first-principles calculation. Adv. Sci. **7**(14), 2000736 (2020). <https://doi.org/10.1002/advs.202000736>
9. D.W. Dees, S. Kawauchi, D.P. Abraham, J. Prakash, Analysis of the Galvanostatic Intermittent Titration Technique (GITT) as applied to a lithium-ion porous electrode. J. Power Sources **189**(1), 263–268 (2009). <https://doi.org/10.1016/j.jpowsour.2008.09.045>
10. T. Li, D. Zhao, M. Shi, T. Wang, Q. Yin et al., MOF-derived N, S Co-doped carbon matrix-encapsulated Cu_2_S nanoparticles as high-performance lithium-ion battery anodes: a joint theoretical and experimental study. J. Mater. Chem. A **11**(3), 1461–1472 (2023). <https://doi.org/10.1039/D2TA08539A>
11. X. Guo, X. Xie, S. Choi, Y. Zhao, H. Liu et al., Sb_2_O_3_/MXene(Ti_3_C_2_T*_x_*) hybrid anode materials with enhanced performance for sodium-ion batteries. J. Mater. Chem. A **5**(24), 12445–12452 (2017). <https://doi.org/10.1039/c7ta02689g>
12. W. Sun, S. Liu, Y. Li, D. Wang, Q. Guo et al., Monodispersed FeS_2_ electrocatalyst anchored to nitrogen-doped carbon host for lithium–sulfur batteries. Adv. Funct. Mater. **32**(43), 2205471 (2022). <https://doi.org/10.1002/adfm.202205471>
13. L. Yan, L.X. Kun, Z.F. Ling, Z.L. Qing, Z. Tao et al., Hollow CoS/C structures for high-performance Li, Na, K ion batteries. Front. Chem. **10**, 845742 (2022). <https://doi.org/10.3389/fchem.2022.845742>
14. G. Jia, D. Chao, N.H. Tiep, Z. Zhang, H.J. Fan, Intercalation Na-ion storage in two-dimensional MoS_2-_*_x_*Sex and capacity enhancement by selenium substitution. Energy Storage Mater. **14**, 136–142 (2018). <https://doi.org/10.1016/j.ensm.2018.02.019>
15. N. Shi, B. Xi, M. Huang, F. Tian, W. Chen et al., One-step construction of MoS_0.74_Se_1.26_/N-doped carbon flower-like hierarchical microspheres with enhanced sodium storage. ACS Appl Mater Interfaces **11**(47), 44342–44351 (2019). <https://doi.org/10.1021/acsami.9b15769>
16. L.Y. Zhen, Z.W. Tao, Y.C. Hao, P.Q. Chang, L.Y. Peng et al., Direct synthesis of FeS/N-doped carbon composite for high-performance sodium-ion batteries. J. Mater. Chem. A **6**, 24702 (2018). <https://doi.org/10.1039/C8TA08562E>
17. Z.Y. Quan, Z.R. Ming, X.Q. Ju, L. Heng, T.M. Li et al., Circuit board-like CoS/MXene composite with superior performance for sodium storage. Chem. Eng. J. **357**, 220-225 (2019). <https://doi.org/10.1016/j.cej.2018.09.142>
18. D. Li, Y. Sun, S. Chen, J. Yao, Y. Zhang et al., Highly porous FeS/carbon fibers derived from Fe-carrageenan biomass: high-capacity and durable anodes for sodium-ion batteries. ACS Appl Mater Interfaces **10**(20), 17175–17182 (2018). <https://doi.org/10.1021/acsami.8b03059>
19. Z.Y. Huang, Y.F. Yuan, Z.J. Yao, M. Zhu, S.M. Yin et al., Metal-organic framework-derived ultrafine CoSe nanocrystal@honeycomb porous carbon nanofiber as multidimensional advanced anode for sodium-ion batteries. Appl. Surf. Sci. **637**, 157886 (2023). <https://doi.org/10.1016/j.apsusc.2023.157886>
20. J. Miao, J. Y. Hong, Z. C. Chen, Z. P. Zhu, J. M. Qiu, High electrochemical sodium storage performance of ZnSe/CoSe@N-doped porous carbon synthesized by the in-situselenization of ZIF-8/67 polyhedron. Appl. Surf. Sci. 2020, 518, 146259. <https://doi.org/10.1016/j.apsusc.2020.146259>
21. M. Wang, H. Xu, Z. Yang, H. Yang, A. Peng et al., SnS nanosheets confined growth by S and N codoped graphene with enhanced pseudocapacitance for sodium-ion capacitors. ACS Appl Mater Interfaces **11**(44), 41363–41373 (2019). <https://doi.org/10.1021/acsami.9b14098>
22. L.-F. Zhou, X.-W. Gao, T. Du, H. Gong, L.-Y. Liu et al., Two-dimensional NbSSe as anode material for low-temperature sodium-ion batteries. Chem. Eng. J. **435**, 134838 (2022). <https://doi.org/10.1016/j.cej.2022.134838>
23. Y. Wang, H. Li, B. Zhai, X. Li, P. Niu et al., Highly crystalline poly(heptazine imide)-based carbonaceous anodes for ultralong lifespan and low-temperature sodium-ion batteries. ACS Nano **18**(4), 3456–3467 (2024). <https://doi.org/10.1021/acsnano.3c10779>
24. L. Liu, S. Li, L. Hu, X. Liang, W. Yang et al., Bi@C nanosphere anode with Na+-ether-solvent cointercalation behavior to achieve fast sodium storage under extreme low temperatures. Carbon Energy **6**(9), e531 (2024). <https://doi.org/10.1002/cey2.531>
25. H. Liang, L. Liu, N. Wang, W. Zhang, C.-T. Hung et al., Unusual mesoporous titanium niobium oxides realizing sodium-ion batteries operated at-40 ℃. Adv. Mater. **34**(28), e2202873 (2022). <https://doi.org/10.1002/adma.202202873>
26. S. Zhang, Z. Cao, P. Fan, Y. Wang, W. Jia et al., A nanopore-based saccharide sensor. Angew. Chem. Int. Ed. **61**(33), e202203769 (2022). <https://doi.org/10.1002/anie.202203769>
27. Y. Qi, J. Li, W. Zhong, S. Bao, M. Xu, KTiOPO_4_: a long-life, high-rate and low-temperature-workable host for Na/K-ion batteries. Chem. Eng. J. **417**, 128159 (2021). <https://doi.org/10.1016/j.cej.2020.128159>
28. M. Jiang, Y. Hu, B. Mao, Y. Wang, Z. Yang et al., Strain-regulated Gibbs free energy enables reversible redox chemistry of chalcogenides for sodium ion batteries. Nat. Commun. **13**(1), 5588 (2022). <https://doi.org/10.1038/s41467-022-33329-2>
29. H. Zhang, J. Song, J. Li, J. Feng, Y. Ma et al., Interlayer-expanded MoS_2_ nanoflowers vertically aligned on MXene@Dual-phased TiO_2_ as high-performance anode for sodium-ion batteries. ACS Appl. Mater. Interfaces **14**(14), 16300–16309 (2022). <https://doi.org/10.1021/acsami.2c02080>
30. X. Rui, X. Zhang, S. Xu, H. Tan, Y. Jiang et al., A low-temperature sodium-ion full battery: superb kinetics and cycling stability. Adv. Funct. Mater. **31**(11), 2009458 (2021). <https://doi.org/10.1002/adfm.202009458>
31. Y. Wang, Y. Kuang, J. Cui, X. Xu, F. Li et al., Self-template construction of hierarchical Bi@C microspheres as competitive wide temperature-operating anodes for superior sodium-ion batteries. Nano Lett. **24**(48), 15242–15251 (2024). <https://doi.org/10.1021/acs.nanolett.4c03453>
32. Y. She, X. Li, Y. Zheng, D. Chen, X. Rui et al., Natural lignin: a sustainable and cost-effective electrode material for high-temperature Na-ion battery. ENERGY ENVIRONMENTAL Mater. **7**(2), e12538 (2024). <https://doi.org/10.1002/eem2.12538>
33. G. Xu, L. Yang, Z. Yan, Z. Huang, X. Li et al., Multiscale structural NaTi_2_(PO4)_3_ anode for sodium-ion batteries with long cycle, high areal capacity, and wide operation temperature. Carbon Energy **6**(10), e552 (2024). <https://doi.org/10.1002/cey2.552>
